# Forensic soil provenancing in an urban/suburban setting: A sequential multivariate approach

**DOI:** 10.1111/1556-4029.14727

**Published:** 2021-05-06

**Authors:** Patrice de Caritat, Brenda Woods, Timothy Simpson, Christopher Nichols, Lissy Hoogenboom, Adriana Ilheo, Michael G. Aberle, Jurian Hoogewerff

**Affiliations:** ^1^ Australian Federal Police Canberra Australian Capital Territory Australia; ^2^ Geoscience Australia Canberra Australian Capital Territory Australia; ^3^ National Centre for Forensic Studies, University of Canberra Bruce Australian Capital Territory Australia; ^4^ Present address: UQ Centre for Natural Gas The University of Queensland St Lucia Queensland Australia; ^5^ Present address: Australian Federal Police Canberra Australian Capital Territory Australia

**Keywords:** compositional data analysis, geochemical mapping, geographic information system, interpolation, soil forensics, soil properties, uncertainty

## Abstract

Compositional data from a soil survey over North Canberra, Australian Capital Territory, are used to develop and test an empirical soil provenancing method. Mineralogical data from Fourier transform infrared spectroscopy (FTIR) and magnetic susceptibility (MS), and geochemical data from X‐ray fluorescence (XRF; for total major oxides) and inductively coupled plasma‐mass spectrometry (ICP‐MS; for both total and *aqua regia*‐soluble trace elements) are performed on the survey's 268 topsoil samples (0–5 cm depth; 1 sample per km^2^). Principal components (PCs) are calculated after imputation of censored data and centered log‐ratio transformation. The sequential provenancing approach is underpinned by (i) the preparation of interpolated raster grids of the soil properties (including PCs); (ii) the explicit quantification and propagation of uncertainty; (iii) the intersection of the soil property rasters with the values of the evidentiary sample (± uncertainty); and (iv) the computation of cumulative provenance rasters (“heat maps”) for the various analytical techniques. The sequential provenancing method is tested on the North Canberra soil survey with three “blind” samples representing simulated evidentiary samples. Performance metrics of precision and accuracy indicate that the FTIR and MS (mineralogy), as well as XRF and total ICP‐MS (geochemistry) analytical methods, offer the most precise and accurate provenance predictions. Inclusion of PCs in provenancing adds marginally to the performance. Maximizing the number of analytes/analytical techniques is advantageous in soil provenancing. Despite acknowledged limitations and gaps, it is concluded that the empirical soil provenancing approach can play an important role in forensic and intelligence applications.

**Highlights**

- Topsoil mineralogical and chemical properties are determined over a 260‐km^2^ area in/around Canberra.
- Those properties are interpolated to create 250 × 250 m raster grids over the survey area.
- Evidentiary (blind) sample properties are compared within uncertainty to grid cell values.
- For every grid cell a score of 1 is given if a property matches the blind sample value, 0 otherwise.
- Scores are added for all properties, mapping areas more closely matching the blind samples.

## 1 INTRODUCTION

Soils are complex mixtures of minerals, amorphous material, organic matter, water, gasses, organisms, and, in places, man‐made particles. The composition of soils is fundamentally controlled by their location through the environmental controls of climate (moisture, temperature), life (plants, organisms), topography (elevation, aspect, slope, relief), substrate (geology, parent material), and time (weathering), among others, as first articulated by Jenny in 1941 [1]. Thus, the natural soil composition varies in a largely predictable and structured, rather than random and chaotic, fashion. Therefore, coherent maps showing the spatial variability of natural soil parameters can be produced provided the density at which they are measured is appropriate relative to the scale of their heterogeneity. Human land use may either confound or complement understanding of the spatial patterns. Once a series of soil property maps are produced, they can serve two important forensic purposes: (i) the evidentiary relevance of observing non‐distinguishable questioned and control samples, and (ii) the potential to constrain the spatial provenance of an unknown questioned soil sample.

The use of geological material such as soil in forensic investigations is increasing in police forces around the world, including the Federal Bureau of Investigation, the Royal Canadian Mounted Police, and the Australian Federal Police (e.g., [2, 3, 4, 5, 6, 7]). In Australia, successful soil forensic investigations have contributed evidence that has been used in Australian Supreme courts (e.g., [8]). Forensic soil provenancing can be defined as the capability to spatially constrain the likely region of origin of an evidentiary sample of earth‐related material [9, 10]. Rawlins et al. [9] characterized the prediction of the provenance of a sample of earth‐related material as “*one of the most difficult and challenging tasks for analytical earth scientists*.*”*

Caritat et al. [11] introduced a predictive soil provenancing method that does not require a specific soil survey to be carried out over an area of interest. More typically, however, forensic soil provenancing is implemented empirically by comparing the spatial multivariate information contained in the evidentiary soil's geochemistry, mineralogy, bulk properties, etc., to either purposely acquired or pre‐existing knowledge (see fig. 1 in [11]). Such knowledge generally is derived from soil geochemical surveys and stored in databases containing this same or similar multivariate information over the region of interest at an appropriate density [12]. Geochemical surveys come in many guises (e.g., [13, 14]) and although many already exist at a range of spatial coverages (continental to local), sampling densities (1 sample per 1000's of km^2^ to 100's of samples per 1 km^2^), and sampling media (materials) selections (topsoil, C horizon, sediment, …), forensic applications have specific requirements that may not have been the primary focus of the original surveys [15]. Despite this, these pre‐existing surveys and associated databases have their use in forensic applications, as long as their limitations (e.g., sampling density, sampling medium, sample collection method) are understood.

Once a database is selected, a number of statistical and visualization analysis tools can be implemented, including univariate, bivariate, and multivariate statistical analysis, exploratory data analysis, analysis of variance, compositional data analysis, spatial interpolation/geostatistics and smoothing, cluster analysis, supervised or unsupervised classification, and data mining (e.g., [16, 17, 18, 19, 20, 21, 22, 23, 24, 25, 26]).

The next step in empirical soil provenancing is the comparison of the evidentiary soil sample's composition with the selected database. Statistical analysis of differences can be performed using a few or many compositional characteristics (including chemical element abundances, isotopes, mineral abundances), their ratios or other calculated indexes, correlation analysis, and/or factor or principal component analysis (e.g., [27, 28, 29, 30, 31, 32, 33, 34]), among others.

Finally, if the evidentiary sample is non‐distinguishable with a particular region of origin, a detailed forensic investigation can proceed there. If unsuccessful or inconclusive, more data and better data must be collated (if pre‐existing) or collected (if not), which may imply undertaking a more refined geochemical survey at a scale relevant for the case at hand.

In this and a companion paper, we describe and compare different approaches to soil provenancing based on a local (i.e., relatively small area and relatively high sampling density) soil geochemical survey in and around North Canberra, Australian Capital Territory, in inland southeastern Australia. The approaches under consideration are (i) a sequential multivariate approach (this paper), and (ii) a simultaneous multivariate (degree of geochemical similarity) approach (upcoming paper in preparation). A complementary probabilistic (likelihood ratio) approach will be published separately (upcoming paper in preparation). The aims of the present contribution accordingly are to:

- briefly introduce the North Canberra soil geochemical survey
- present the sequential multivariate provenancing approach
- present results for this method
- quantify the performance of this approach
- draw conclusions as to the suitability of the sequential multivariate provenancing approach for forensic and intelligence applications

## 2 MATERIALS AND METHODS

### 2.1 The North Canberra soil geochemical survey

**FIGURE 1:**
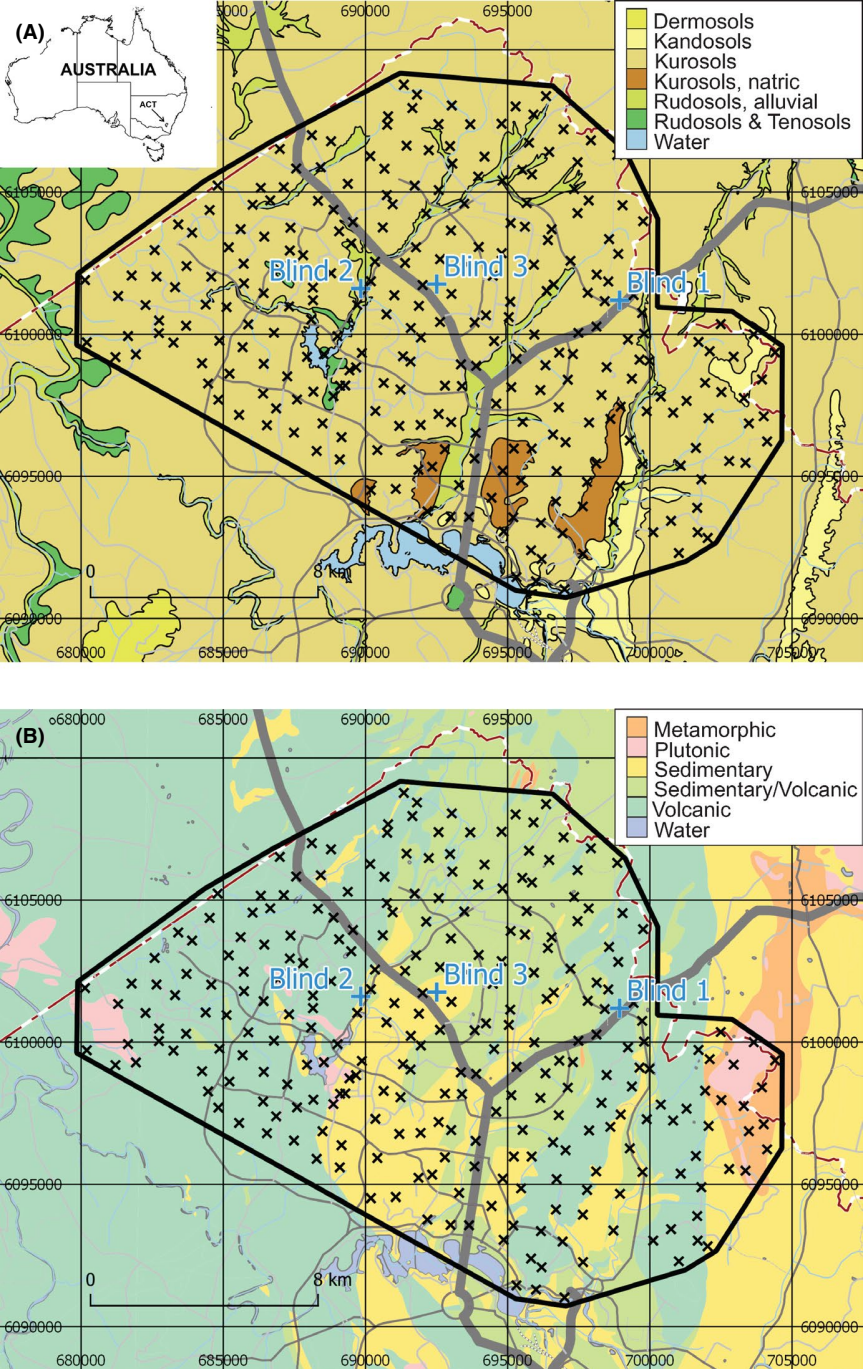
Sample locations (crosses) for the North Canberra, Australian Capital Territory (ACT), soil geochemical survey overlain on Australian Soil Classification (ASC) soil orders [36] (A), and high‐order lithology types (geology) (B). Study area outlined in solid black line. Principal, main, and secondary roads are shown as thick, thin, and dashed gray lines, respectively. Water bodies and drainage are shown in blue. ACT border is shown as brown dash‐dotted line. Geospatial data from ACT Government or Australian Government, unless otherwise indicated [Color figure can be viewed at wileyonlinelibrary.com]

The North Canberra soil geochemical survey was initiated in 2017 and focused on the northern part of Canberra city and surrounding suburban areas, in the Australian Capital Territory (ACT) (Figure 1). The total area covered by the survey was ~260 km^2^ sampled at an average density of 1 site/km^2^ [35]. In addition to the 268 samples in this survey, three “blind” samples (Blind 1, Blind 2, and Blind 3 hereafter) were collected from sites within the survey area (but away from the survey's grid samples), the geographical coordinates or even approximate locations of which were unknown to the lead researcher until the project had concluded all data analysis and map production.

General background, results, and interpretations of the geochemical mapping of the ACT, including the investigation of the effects of lithology and land use on soil geochemistry, will be presented elsewhere (upcoming paper in preparation). A simple description of the blind sample sites is, however, warranted here as it will have a bearing on the interpretation of the provenance analysis we focus on (see Fig. S1 in Appendix S1). Blind 1 is a Kurosol soil (all soil types from the Australian Soil Classification (ASC) [36]) collected over the Mount Ainslie Volcanics Formation, a Wenlockian (Early Silurian) dacitic ignimbrite with minor ashstone, agglomerate and shale, and porphyry, within the Hawkins Volcanic Suite (all stratigraphic units from the Australian Stratigraphic Units Database https://asud.ga.gov.au/). We note here that Blind 1 was deliberately collected from a local environment not representative of the broader landscape to test the limit of soil provenancing. Blind 2 is Kurosol/Rudosol (Alluvial) soil collected over undifferentiated Quaternary alluvium and fluvial deposits of gravel, sand, silt, and clay along Ginninderra Creek. Blind 3 is a Kurosol soil collected over a thin, folded Acton Shale Member, an Early Ordovician black graptolitic siliceous shale within the broader turbiditic (sandstone, mudstone, shale) Adaminaby Group.

In this paper, the analytical focus is directed to both (i) soil mineralogy via infrared spectroscopy (informing on, e.g., hydrated minerals such as clay minerals, carbonates, and sulfates) and magnetic susceptibility (informing on, e.g., ferrimagnetic minerals such as maghemite or magnetite, and their grain sizes); and (ii) soil geochemistry via major oxides and organic matter concentrations as well as trace element concentrations after two chemical extractions of different strengths. Sample collection, preparation, and analysis methods are detailed in the Appendix S1, as are data analysis, spatial analysis, quality control, and detailed uncertainty analysis procedures.

### 2.2 Uncertainty analysis

Uncertainty arises from any attempt to quantify natural phenomena, from sampling through to analysis. In this project, two main types of uncertainty were specifically quantified: measurement uncertainty (*U*_m_) and interpolation uncertainty (*U*_i_). They were quantified as three standard deviations of field triplicates (SD_m_) and of residuals (SD_i_), respectively. Residuals are the differences between the interpolated (modeled) values and the measured values at each sampled site. The combined uncertainty (*U*_c_), which applies to the generated property raster surfaces, is calculated using the root sum of squares method (e.g., [37, 38]) as follows:

(1) $$U_{c}=\sqrt{{\left(U_{m}\right)}^{2}+{\left(U_{i}\right)}^{2}}=\sqrt{{\left(3\times {\text{SD}}_{m}\right)}^{2}+{\left(3\times {\text{SD}}_{i}\right)}^{2}}$$

The standard deviations (SD_m_, SD_i_) and uncertainties (*U*_m_, *U*_i_, *U*_c_) of each analyte are given in Table S3 in the Appendix S1.

### 2.3 Determination of search ranges

For each variable, the Search Range (SR) for a blind (evidentiary) sample was set to the measured value of that variable in that blind sample (Target Value or TV) with a buffer reflecting the sum of the uncertainty in the analytical data (*U*_m_) and of the uncertainty in the raster surface (*U*_c_), according to:

(2) $$\text{SR}=\text{TV}\pm (U_{m}+U_{c})$$

**FIGURE 2:**
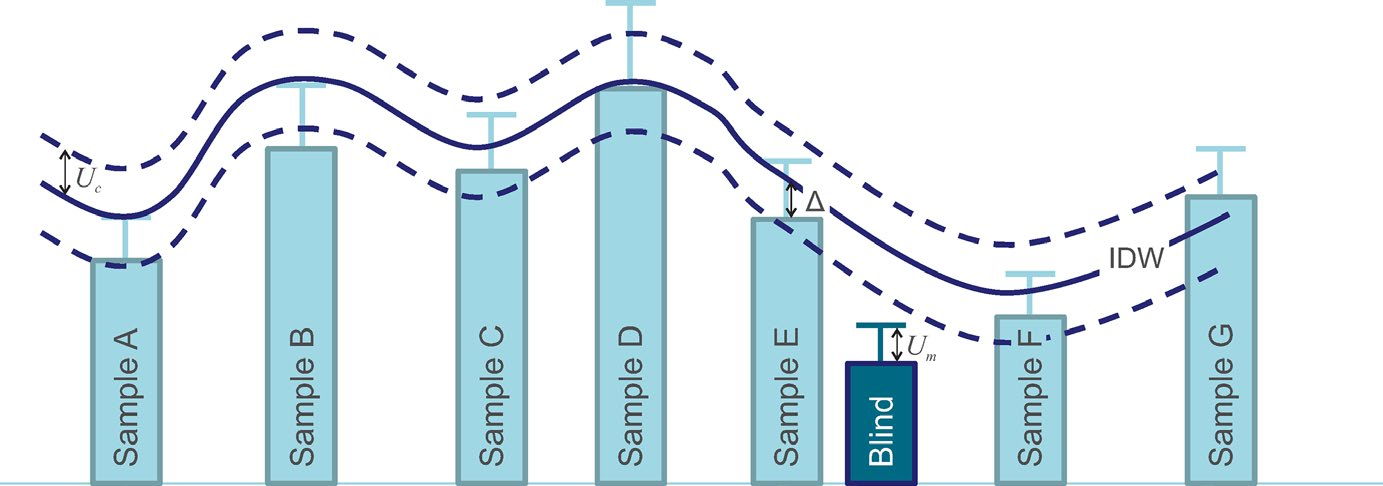
Schematic illustration of the values of a measured variable at seven survey samples A to G (light blue rectangles) with uncertainty (light blue error bars), and inverse distance weighting (IDW) interpolated surface (solid dark blue line) with combined uncertainty *U*_c_ (dashed dark blue lines above and below solid line). Blind sample being provenanced is shown as a dark blue rectangle, with its measurement uncertainty *U*_m_ (dark blue error bar) [Color figure can be viewed at wileyonlinelibrary.com]

This accounts for uncertainty in both the interpolated surface (which is derived from measured values in the database and a smoothing interpolation algorithm), via *U*_c_, and the measured value in the blind sample, via *U*_m_, as illustrated in Figure 2. The graphic illustrates that the interpolated grid value for a particular soil property needs to fall within the uncertainty envelope (*U*_m_ + *U*_c_) around that soil property for the evidentiary sample to count as a match and score a 1 in the provenance raster computation (see below and Appendix S1).

### 2.4 Raster generation and clipping

Interpolation rasters for each available variable were prepared by inverse distance weighting (IDW), clipped, and analyzed in QGIS as explained in the Spatial Analysis section of the Appendix S1.

### 2.5 Provenancing methodology

A sequential multivariate approach to soil provenancing based on an empirical database of soil properties is developed in this contribution. The first step in this approach is to measure and map a number of mineralogical (e.g., FTIR, MS) and geochemical (e.g., XRF and ICP‐MS) soil properties at the sampled sites. The next step is the interpolation of those properties between sampled sites, here performed using IDW (power 3; 12 neighbors; 250 m cells) as detailed elsewhere. The final step of this method is to select raster cells from those grids that match the Target Value ± Search Range of the evidentiary sample of interest. This is akin to drawing contours on a topographic map that follow a given elevation with allowance for some slack or uncertainty in that elevation value; this essentially yields a corridor (or corridors) of locations (cells) that satisfy the elevation ± uncertainty criterion. A raster calculation in QGIS assigns a value of 1 to cells that satisfy a given criterion (i.e., those whose soil property value fall within the Search Range), and a value of 0 to those that do not (i.e., those whose soil property value fall outside the Search Range). Once the cells that satisfy the Search Range for one composition variable are established, those for one or many more variables can be added to it. This generates a map over the area of interest with cells having values ranging from 0 to N (the number of soil properties under consideration). Such maps can be colored to produce “heat maps” that readily draw attention to those areas with most criteria being satisfied and thus more likely to include the potential origin for the evidentiary sample. It is noted that the provenancing methodology presented here is not intended to be used at the exclusion of other provenancing avenues such as soil microbiome or palynology, but rather complement those by providing a geochemical/mineralogical perspective. Once areas of enhanced provenance potential are identified, further resources can be allocated to these focussed regions with a lower failure risk.

## 3 RESULTS AND DISCUSSION

A statistical summary of the data collected during this project can be found in Table 1. Lower limits of detection and proportions of the variance explained for the principal components obtained for the FTIR, XRF, Total, and *aqua regia* (AR) ICP‐MS datasets (the latter three after centered log‐ratio—clr—transformation) are given in the Appendix S1 (Tables S1 and S2).

**TABLE 1:** Statistical summary (minimum, median, average, maximum, and standard deviation) of the compositional and/or principal components (PCs) variables for the North Canberra soils including the three blind samples

Variable	Min	Med	Ave	Max	SD	Variable	Min	Med	Ave	Max	SD
PC1_FTIR	−6.314	−0.101	−0.221	3.435	1.701	As_Tot	0.83	3.55	4.77	35.7	4.24
PC2_FTIR	−1.455	−0.026	0.011	1.339	0.523	Ba_Tot	126	311	325	1100	107
PC3_FTIR	−0.727	0.005	0.001	0.975	0.235	Bi_Tot	<0.06	0.30	0.40	4.70	0.46
Xlf	0.11	0.56	0.85	34.1	2.13	Ce_Tot	37.1	67.8	69.3	174	17.2
Xfd_pc	0.22	9.04	8.95	13.0	1.91	Co_Tot	1.69	8.24	8.61	44.0	4.00
SiO2_XRF	54.4	75.2	75.1	87.6	5.2	Cr_Tot	22.8	46.8	48.1	123	11.6
TiO2_XRF	0.63	0.91	0.92	2.00	0.12	Cs_Tot	1.17	2.93	3.20	12.5	1.45
Al2O3_XRF	3.55	9.07	9.14	17.6	2.12	Cu_Tot	8.70	19.9	22.1	274	17.06
Fe2O3_XRF	1.11	2.63	2.79	17.8	1.25	Dy_Tot	3.00	5.03	5.11	13.5	1.15
MnO_XRF	0.01	0.07	0.07	0.45	0.04	Er_Tot	1.94	3.15	3.23	9.86	0.70
MgO_XRF	0.11	0.46	0.52	1.71	0.29	Eu_Tot	0.50	0.93	0.95	2.62	0.27
CaO_XRF	0.05	0.31	0.37	1.24	0.22	Ga_Tot	4.50	10.6	10.9	21.0	2.66
Na2O_XRF	0.17	0.73	0.80	4.53	0.50	Gd_Tot	2.85	4.95	5.09	13.7	1.30
K2O_XRF	0.66	1.69	1.74	4.10	0.63	Ge_Tot	0.99	1.37	1.39	7.01	0.38
P2O5_XRF	0.04	0.09	0.10	0.24	0.03	Hf_Tot	4.55	14.7	14.9	40.7	4.31
LOI_XRF	3.85	7.85	8.04	14.4	2.16	Ho_Tot	0.63	1.04	1.06	3.02	0.23
PC1_XRF	−6.13	−0.15	−0.12	6.15	2.01	La_Tot	18.1	33.1	34.3	85.0	8.6
PC2_XRF	−4.93	0.14	0.28	5.61	1.78	Lu_Tot	0.29	0.51	0.52	1.73	0.11
PC3_XRF	−8.93	−0.12	−0.22	4.35	1.74	Nb_Tot	10.0	14.8	15.0	30.8	2.16
PC4_XRF	−3.64	−0.05	−0.07	4.58	1.31	Nd_Tot	15.4	28.1	28.9	73.4	7.53
PC5_XRF	−5.08	0.02	−0.01	4.18	1.37	Ni_Tot	<2.40	14.7	15.2	33.4	5.04
PC6_XRF	−1.70	0.03	0.11	2.98	0.92	Pb_Tot	6.71	19.1	23.7	292	21.1
PC7_XRF	−3.68	−0.08	−0.03	3.57	0.92	Pr_Tot	4.15	7.66	7.92	20.2	2.07
PC8_XRF	−2.73	−0.05	−0.03	2.07	0.69	Rb_Tot	33.7	83.0	86.0	201	26.2
Ba_AR	16.5	71.3	72.2	162	24.5	Sc_Tot	4.42	9.72	9.88	19.6	2.46
Be_AR	0.15	0.55	0.57	1.48	0.21	Sm_Tot	2.82	5.35	5.47	14.5	1.48
Ce_AR	9.51	38.7	38.6	120	13.9	Sn_Tot	1.65	3.06	3.25	15.5	1.17
Co_AR	0.74	7.88	8.30	44.9	4.42	Sr_Tot	21.6	51.4	54.3	133	16.7
Cs_AR	<0.30	0.58	0.67	5.14	0.42	Ta_Tot	0.85	1.26	1.28	2.88	0.20
Dy_AR	0.33	1.66	1.76	9.51	0.95	Tb_Tot	0.48	0.84	0.86	2.16	0.21
Er_AR	0.14	0.78	0.83	4.40	0.47	Th_Tot	8.36	13.3	13.7	35.5	3.00
Eu_AR	0.13	0.57	0.60	2.57	0.30	U_Tot	2.18	3.12	3.23	6.97	0.58
Fe_AR	4742	13081	14269	130186	8813	V_Tot	22.5	60.9	62.4	209	17.4
La_AR	5.10	18.0	18.6	58.5	7.15	W_Tot	1.69	2.53	2.78	10.7	0.95
Mn_AR	17.0	432	463	2984	320	Y_Tot	16.8	28.3	28.9	82.4	6.3
Nd_AR	4.02	17.1	17.4	61.7	7.34	Yb_Tot	2.06	3.29	3.36	11.1	0.72
Ni_AR	1.86	8.32	9.11	28.8	4.49	Zn_Tot	12.9	44.6	50.8	315	31.3
Pb_AR	3.71	15.3	20.4	394	27.0	Zr_Tot	175	564	579	1621	174
Rb_AR	4.35	11.5	13.0	33.3	5.34	PC1_Tot	−10.567	−0.216	0.035	23.291	4.385
Sr_AR	1.84	12.7	13.9	40.0	6.69	PC2_Tot	−14.872	0.065	0.143	9.352	3.750
Th_AR	0.52	1.92	2.19	16.1	1.38	PC3_Tot	−10.272	−0.047	−0.506	4.520	2.468
Tm_AR	0.01	0.09	0.10	0.49	0.06	PC4_Tot	−6.613	0.151	0.122	12.971	2.365
Zn_AR	<15.0	43.7	51.0	369	35.1	PC5_Tot	−4.082	0.114	0.195	7.237	1.811
PC1_AR	−10.009	−0.242	−0.138	7.409	2.338	PC6_Tot	−6.451	−0.066	0.013	6.767	1.866
PC2_AR	−6.681	0.343	0.016	3.211	1.719	PC7_Tot	−4.621	−0.154	−0.259	4.564	1.563
PC3_AR	−6.217	0.046	0.000	3.458	1.366	PC8_Tot	−6.424	0.140	0.140	4.334	1.462
PC4_AR	−3.845	−0.052	−0.082	3.493	1.227						
PC5_AR	−4.227	0.094	0.036	3.675	1.128						
PC6_AR	−3.077	0.122	0.033	2.708	1.029						
PC7_AR	−2.799	−0.012	0.000	2.828	0.873						
PC8_AR	−3.315	0.095	0.029	2.463	0.799						

Methods are as follows: Fourier transform infrared (_FTIR), mass‐specific (Xlf) and frequency‐dependent in percent (Xfd_pc) magnetic susceptibility, X‐ray fluorescence (_XRF), and aqua regia (_AR) and total (_Tot) inductively coupled plasma‐mass spectrometry. Units are as follows: All PCs: dimensionless; Xlf: 10^−6^ m^3^/kg; Xfd_pc: %; XRF: wt%; AR and Tot: mg/kg (ppm). See text for details.

### 3.1 Validation

Standard deviations and uncertainties derived for each parameter as described above are given in the Appendix S1 (Table S3). The Target Values and Search Ranges for the Blind 1, Blind 2, and Blind 3 evidentiary samples collected as part of this project are shown in Tables 2, 3, and 4, respectively. The results of soil provenancing investigations using the sequential multivariate approach are discussed below.

**TABLE 2:** Target Values (TV) and Search Ranges (SR) for unknown sample Blind 1 for all variables

Variable	TV	SR From	SR To	Variable	TV	SR From	SR To
PC1_FTIR	2.034	0.124	3.943	As_Tot	1.78	0.62	2.94
PC2_FTIR	0.013	−0.099	0.125	Ba_Tot	373	296	450
PC3_FTIR	−0.106	−0.355	0.143	Bi_Tot	0.12	−0.20	0.44
Xlf	0.35	−0.25	0.95	Ce_Tot	46.5	36.5	56.5
Xfd_pc	9.74	8.53	11.0	Co_Tot	6.52	4.41	8.64
SiO2_XRF	76.0	73.9	78.2	Cr_Tot	36.4	25.9	47.0
TiO2_XRF	1.00	0.94	1.05	Cs_Tot	1.83	0.84	2.83
Al2O3_XRF	8.34	7.42	9.26	Cu_Tot	15.4	−17.0	47.8
Fe2O3_XRF	2.19	1.31	3.08	Dy_Tot	4.00	3.25	4.74
MnO_XRF	0.04	0.02	0.06	Er_Tot	2.67	2.27	3.06
MgO_XRF	0.83	0.66	1.01	Eu_Tot	0.73	0.56	0.89
CaO_XRF	0.15	−0.02	0.33	Ga_Tot	8.97	6.79	11.1
Na2O_XRF	1.05	0.95	1.14	Gd_Tot	3.53	2.84	4.21
K2O_XRF	1.92	1.69	2.14	Ge_Tot	1.21	0.93	1.50
P2O5_XRF	0.09	0.07	0.10	Hf_Tot	15.3	13.3	17.2
LOI_XRF	8.11	6.59	9.62	Ho_Tot	0.85	0.71	0.98
PC1_XRF	−0.403	−1.313	0.507	La_Tot	24.2	17.5	30.8
PC2_XRF	1.513	0.871	2.155	Lu_Tot	0.47	0.40	0.53
PC3_XRF	0.474	−0.223	1.171	Nb_Tot	15.9	14.3	17.4
PC4_XRF	−1.566	−2.423	−0.710	Nd_Tot	18.9	14.4	23.4
PC5_XRF	0.620	0.111	1.128	Ni_Tot	12.4	5.83	19.0
PC6_XRF	−1.087	−1.824	−0.349	Pb_Tot	14.1	−0.67	28.9
PC7_XRF	−0.544	−0.996	−0.092	Pr_Tot	5.19	3.97	6.41
PC8_XRF	−1.334	−1.726	−0.942	Rb_Tot	81.5	62.9	100
Ba_AR	40.2	22.2	58.2	Sc_Tot	12.7	11.0	14.3
Be_AR	0.37	0.19	0.54	Sm_Tot	3.56	2.53	4.58
Ce_AR	21.5	11.9	31.1	Sn_Tot	1.97	1.37	2.58
Co_AR	5.48	1.84	9.12	Sr_Tot	53.9	45.5	62.3
Cs_AR	0.44	0.23	0.64	Ta_Tot	1.37	1.22	1.51
Dy_AR	0.93	0.26	1.60	Tb_Tot	0.61	0.42	0.79
Er_AR	0.42	0.10	0.74	Th_Tot	10.7	8.67	12.7
Eu_AR	0.33	0.09	0.57	U_Tot	2.95	2.60	3.30
Fe_AR	10672	5581	15764	V_Tot	61.5	45.2	77.8
La_AR	11.6	7.70	15.5	W_Tot	3.27	2.97	3.57
Mn_AR	237	16.7	457	Y_Tot	22.9	18.7	27.0
Nd_AR	8.77	3.30	14.2	Yb_Tot	2.88	2.45	3.31
Ni_AR	3.80	1.35	6.25	Zn_Tot	45.0	32.8	57.2
Pb_AR	9.45	−5.32	24.2	Zr_Tot	613	519	707
Rb_AR	12.2	9.41	15.0	PC1_Tot	−0.052	−4.558	4.455
Sr_AR	5.89	0.32	11.5	PC2_Tot	4.131	1.479	6.783
Th_AR	1.97	1.37	2.56	PC3_Tot	−4.094	−7.734	−0.455
Tm_AR	0.05	0.02	0.08	PC4_Tot	−1.648	−3.217	−0.078
Zn_AR	28.9	2.34	55.5	PC5_Tot	0.783	−1.693	3.259
PC1_AR	0.653	−1.282	2.588	PC6_Tot	−0.178	−1.463	1.106
PC2_AR	−0.674	−1.273	−0.075	PC7_Tot	1.527	0.796	2.259
PC3_AR	0.278	−0.688	1.245	PC8_Tot	−1.045	−3.064	0.974
PC4_AR	0.236	−0.772	1.245				
PC5_AR	1.117	0.565	1.669				
PC6_AR	−1.062	−2.276	0.152				
PC7_AR	0.679	−0.007	1.365				
PC8_AR	0.831	0.230	1.432				

Methods are as follows: Fourier transform infrared (_FTIR), mass‐specific (Xlf) and frequency‐dependent in percent (Xfd_pc) magnetic susceptibility, X‐ray fluorescence (_XRF), and aqua regia (_AR) and total (_Tot) inductively coupled plasma‐mass spectrometry. Units are as follows: All PCs: dimensionless; Xlf: 10^−6^ m^3^/kg; Xfd_pc: %; XRF: wt%; AR and Tot: mg/kg (ppm). See text for details.

**TABLE 3:** Target Values (TV) and Search Ranges (SR) for unknown sample Blind 2 for all variables

Variable	TV	SR From	SR To	Variable	TV	SR From	SR To
PC1_FTIR	−1.481	−3.390	0.429	As_Tot	4.72	3.55	5.88
PC2_FTIR	−0.556	−0.667	−0.444	Ba_Tot	306	229	383
PC3_FTIR	−0.220	−0.469	0.029	Bi_Tot	0.36	0.04	0.68
Xlf	0.24	−0.36	0.83	Ce_Tot	66.1	56.1	76.1
Xfd_pc	2.04	0.83	3.25	Co_Tot	8.15	6.04	10.3
SiO2_XRF	69.6	67.5	71.7	Cr_Tot	55.9	45.3	66.5
TiO2_XRF	0.80	0.74	0.85	Cs_Tot	3.86	2.87	4.86
Al2O3_XRF	10.9	9.98	11.8	Cu_Tot	22.5	−9.92	54.9
Fe2O3_XRF	3.50	2.62	4.39	Dy_Tot	5.03	4.28	5.77
MnO_XRF	0.05	0.02	0.07	Er_Tot	3.02	2.62	3.41
MgO_XRF	0.96	0.79	1.14	Eu_Tot	1.01	0.84	1.18
CaO_XRF	0.84	0.66	1.01	Ga_Tot	13.0	10.8	15.2
Na2O_XRF	0.68	0.58	0.77	Gd_Tot	5.08	4.39	5.77
K2O_XRF	1.79	1.57	2.01	Ge_Tot	1.58	1.29	1.86
P2O5_XRF	0.10	0.08	0.11	Hf_Tot	10.8	8.87	12.7
LOI_XRF	10.6	9.04	12.1	Ho_Tot	1.01	0.87	1.15
PC1_XRF	−2.884	−3.794	−1.974	La_Tot	32.3	25.6	38.9
PC2_XRF	−0.392	−1.033	0.250	Lu_Tot	0.46	0.40	0.53
PC3_XRF	−0.998	−1.695	−0.301	Nb_Tot	12.7	11.1	14.3
PC4_XRF	0.247	−0.609	1.104	Nd_Tot	28.7	24.2	33.2
PC5_XRF	0.873	0.364	1.382	Ni_Tot	19.3	12.8	25.9
PC6_XRF	0.497	−0.241	1.234	Pb_Tot	25.0	10.2	39.8
PC7_XRF	1.633	1.181	2.085	Pr_Tot	7.72	6.50	8.93
PC8_XRF	−0.860	−1.253	−0.468	Rb_Tot	98.8	80.2	117
Ba_AR	76.1	58.1	94.1	Sc_Tot	11.4	9.72	13.0
Be_AR	0.67	0.49	0.85	Sm_Tot	5.50	4.48	6.52
Ce_AR	37.8	28.2	47.4	Sn_Tot	2.93	2.33	3.54
Co_AR	7.90	4.26	11.5	Sr_Tot	72.3	63.9	80.7
Cs_AR	0.56	0.36	0.76	Ta_Tot	1.08	0.94	1.22
Dy_AR	2.17	1.49	2.84	Tb_Tot	0.81	0.63	0.99
Er_AR	1.07	0.75	1.39	Th_Tot	13.1	11.1	15.1
Eu_AR	0.74	0.50	0.98	U_Tot	2.79	2.44	3.14
Fe_AR	16685	11593	21777	V_Tot	70.1	53.8	86.4
La_AR	18.0	14.1	21.9	W_Tot	2.09	1.79	2.39
Mn_AR	315	95.1	535	Y_Tot	26.7	22.5	30.9
Nd_AR	18.7	13.2	24.1	Yb_Tot	3.00	2.57	3.42
Ni_AR	13.2	10.7	15.6	Zn_Tot	72.4	60.1	84.6
Pb_AR	21.0	6.20	35.8	Zr_Tot	421	328	515
Rb_AR	10.4	7.64	13.2	PC1_Tot	−3.842	−8.348	0.664
Sr_AR	35.5	30.0	41.1	PC2_Tot	−1.444	−4.096	1.208
Th_AR	2.10	1.51	2.70	PC3_Tot	−0.945	−4.584	2.695
Tm_AR	0.13	0.10	0.17	PC4_Tot	0.456	−1.113	2.025
Zn_AR	73.27	46.68	99.87	PC5_Tot	−0.165	−2.642	2.311
PC1_AR	0.082	−1.853	2.017	PC6_Tot	0.986	−0.298	2.270
PC2_AR	0.955	0.356	1.554	PC7_Tot	0.900	0.168	1.632
PC3_AR	−0.731	−1.698	0.236	PC8_Tot	−0.099	−2.118	1.920
PC4_AR	0.969	−0.039	1.978				
PC5_AR	−0.357	−0.908	0.195				
PC6_AR	2.708	1.494	3.922				
PC7_AR	0.045	−0.641	0.731				
PC8_AR	−0.394	−0.995	0.206				

Methods are as follows: Fourier transform infrared (_FTIR), mass‐specific (Xlf) and frequency‐dependent in percent (Xfd_pc) magnetic susceptibility, X‐ray fluorescence (_XRF), and aqua regia (_AR) and total (_Tot) inductively coupled plasma‐mass spectrometry. Units are as follows: All PCs: dimensionless; Xlf: 10^−6^ m^3^/kg; Xfd_pc: %; XRF: wt%; AR and Tot: mg/kg (ppm). See text for details.

**TABLE 4:** Target Values (TV) and Search Ranges (SR) for unknown sample Blind 3 for all variables

Variable	TV	SR From	SR To	Variable	TV	SR From	SR To
PC1_FTIR	2.656	0.746	4.565	As_Tot	3.45	2.29	4.61
PC2_FTIR	0.644	0.532	0.756	Ba_Tot	248	171	326
PC3_FTIR	0.135	−0.115	0.384	Bi_Tot	0.22	−0.10	0.54
Xlf	0.37	−0.23	0.97	Ce_Tot	48.0	38.0	58.0
Xfd_pc	10.2	9.02	11.4	Co_Tot	2.38	0.26	4.49
SiO2_XRF	83.4	81.3	85.6	Cr_Tot	44.2	33.7	54.8
TiO2_XRF	0.90	0.85	0.95	Cs_Tot	2.00	1.00	2.99
Al2O3_XRF	4.89	3.97	5.81	Cu_Tot	17.9	−14.5	50.3
Fe2O3_XRF	1.63	0.74	2.51	Dy_Tot	4.10	3.35	4.84
MnO_XRF	0.03	0.00	0.05	Er_Tot	2.78	2.39	3.18
MgO_XRF	0.21	0.03	0.38	Eu_Tot	0.55	0.39	0.72
CaO_XRF	0.17	−0.01	0.34	Ga_Tot	6.13	3.95	8.31
Na2O_XRF	0.22	0.12	0.31	Gd_Tot	3.54	2.85	4.23
K2O_XRF	0.84	0.62	1.07	Ge_Tot	1.37	1.08	1.66
P2O5_XRF	0.09	0.07	0.10	Hf_Tot	20.0	18.0	21.9
LOI_XRF	7.43	5.92	8.94	Ho_Tot	0.87	0.74	1.01
PC1_XRF	3.842	2.932	4.752	La_Tot	24.3	17.7	31.0
PC2_XRF	−1.175	−1.817	−0.534	Lu_Tot	0.51	0.45	0.58
PC3_XRF	−1.633	−2.330	−0.936	Nb_Tot	14.6	13.1	16.2
PC4_XRF	−1.417	−2.273	−0.560	Nd_Tot	19.5	15.0	24.0
PC5_XRF	1.414	0.905	1.922	Ni_Tot	10.4	3.83	17.0
PC6_XRF	0.527	−0.211	1.264	Pb_Tot	12.6	−2.21	27.4
PC7_XRF	0.905	0.453	1.357	Pr_Tot	5.40	4.19	6.62
PC8_XRF	0.594	0.202	0.987	Rb_Tot	46.7	28.2	65.3
Ba_AR	52.4	34.4	70.4	Sc_Tot	6.28	4.62	7.93
Be_AR	0.27	0.09	0.45	Sm_Tot	3.62	2.60	4.64
Ce_AR	12.4	2.80	22.0	Sn_Tot	2.07	1.47	2.68
Co_AR	1.64	−2.00	5.28	Sr_Tot	34.0	25.6	42.4
Cs_AR	0.48	0.28	0.69	Ta_Tot	1.20	1.06	1.35
Dy_AR	0.36	−0.31	1.04	Tb_Tot	0.60	0.41	0.78
Er_AR	0.15	−0.17	0.47	Th_Tot	11.3	9.34	13.3
Eu_AR	0.16	−0.08	0.40	U_Tot	3.06	2.71	3.41
Fe_AR	10923	5832	16015	V_Tot	65.9	49.5	82.2
La_AR	5.98	2.06	9.89	W_Tot	2.06	1.76	2.36
Mn_AR	179	−41.3	399	Y_Tot	24.3	20.1	28.5
Nd_AR	5.00	−0.47	10.5	Yb_Tot	3.17	2.74	3.59
Ni_AR	4.74	2.30	7.19	Zn_Tot	17.6	5.37	29.8
Pb_AR	10.6	−4.15	25.4	Zr_Tot	807	713	900
Rb_AR	8.31	5.53	11.1	PC1_Tot	4.123	−0.383	8.629
Sr_AR	15.1	9.54	20.7	PC2_Tot	5.258	2.606	7.910
Th_AR	0.98	0.38	1.58	PC3_Tot	−1.376	−5.015	2.263
Tm_AR	0.02	−0.01	0.05	PC4_Tot	0.694	−0.875	2.263
Zn_AR	21.9	−4.67	48.5	PC5_Tot	−3.347	−5.823	−0.870
PC1_AR	6.347	4.412	8.282	PC6_Tot	1.585	0.301	2.869
PC2_AR	−2.496	−3.095	−1.897	PC7_Tot	−1.328	−2.059	−0.596
PC3_AR	−0.490	−1.456	0.477	PC8_Tot	0.043	−1.976	2.061
PC4_AR	−1.894	−2.902	−0.885				
PC5_AR	−0.194	−0.746	0.358				
PC6_AR	0.616	−0.598	1.829				
PC7_AR	0.178	−0.507	0.864				
PC8_AR	0.568	−0.033	1.169				

Methods are as follows: Fourier transform infrared (_FTIR), mass‐specific (Xlf) and frequency‐dependent in percent (Xfd_pc) magnetic susceptibility, X‐ray fluorescence (_XRF), and aqua regia (_AR) and total (_Tot) inductively coupled plasma‐mass spectrometry. Units are as follows: All PCs: dimensionless; Xlf: 10^−6^ m^3^/kg; Xfd_pc: %; XRF: wt%; AR and Tot: mg/kg (ppm). See text for details.

**FIGURE 3:**
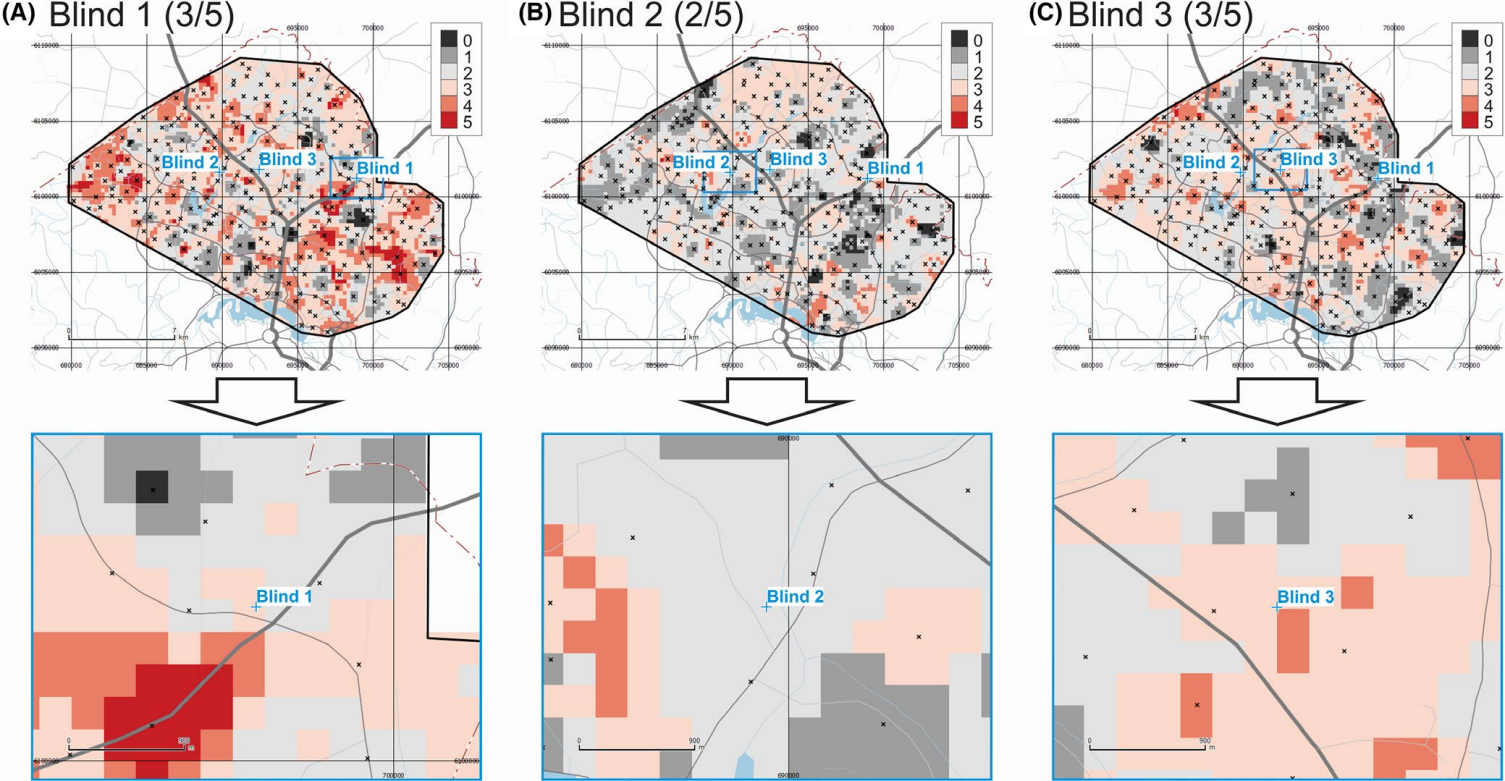
Provenance prediction maps for unknown samples Blind 1 (A), Blind 2 (B), and Blind 3 (C) based on FTIR PC1 to PC3, and MS Xlf and Xfd_pc parameters (overview on top, detail below). Each raster cell gets a score of 1 if it satisfies the Search Range for any of these parameters, and 0 if not. Maximum theoretical score = 5. Geospatial data from ACT Government or Australian Government, unless otherwise indicated [Color figure can be viewed at wileyonlinelibrary.com]

The maps of provenance prediction for samples Blind 1, Blind 2, and Blind 3 based on three FTIR principal components and two MS parameters (for a total of five parameters) are shown in Figure 3. Results indicate that for these three blind samples, 3 of (a theoretical maximum of) 5, 2 of 5, and 3 of 5 parameters match the Search Ranges for Blind 1, Blind 2, and Blind 3, respectively. If the three PCs from FTIR are removed from the analysis and only MS data are considered (not shown), the match rates for these three blind samples change to 1 of 2 for all three blind samples.

The soil provenance rasters generated by the present sequential multivariate provenancing method can be interpreted like “heat maps” where raster grid cells with hotter colors are a better match to the evidentiary sample under investigation than cooler colored cells. In Figure 3A, grid cells colored light, medium, and dark red (scores of 3, 4, or 5) indicate a match equivalent or superior to the cell from which simulated evidentiary sample Blind 1 actually comes from (which has score of 3). Provenancing grids computed from the cumulative results from more variables yield smoother, more gradational spatial patterns than those generated from fewer variables, as demonstrated by subsequent figures. In a separate section (Performance Assessment), we will discuss metrics to quantify how good the provenance predictions are.

**FIGURE 4:**
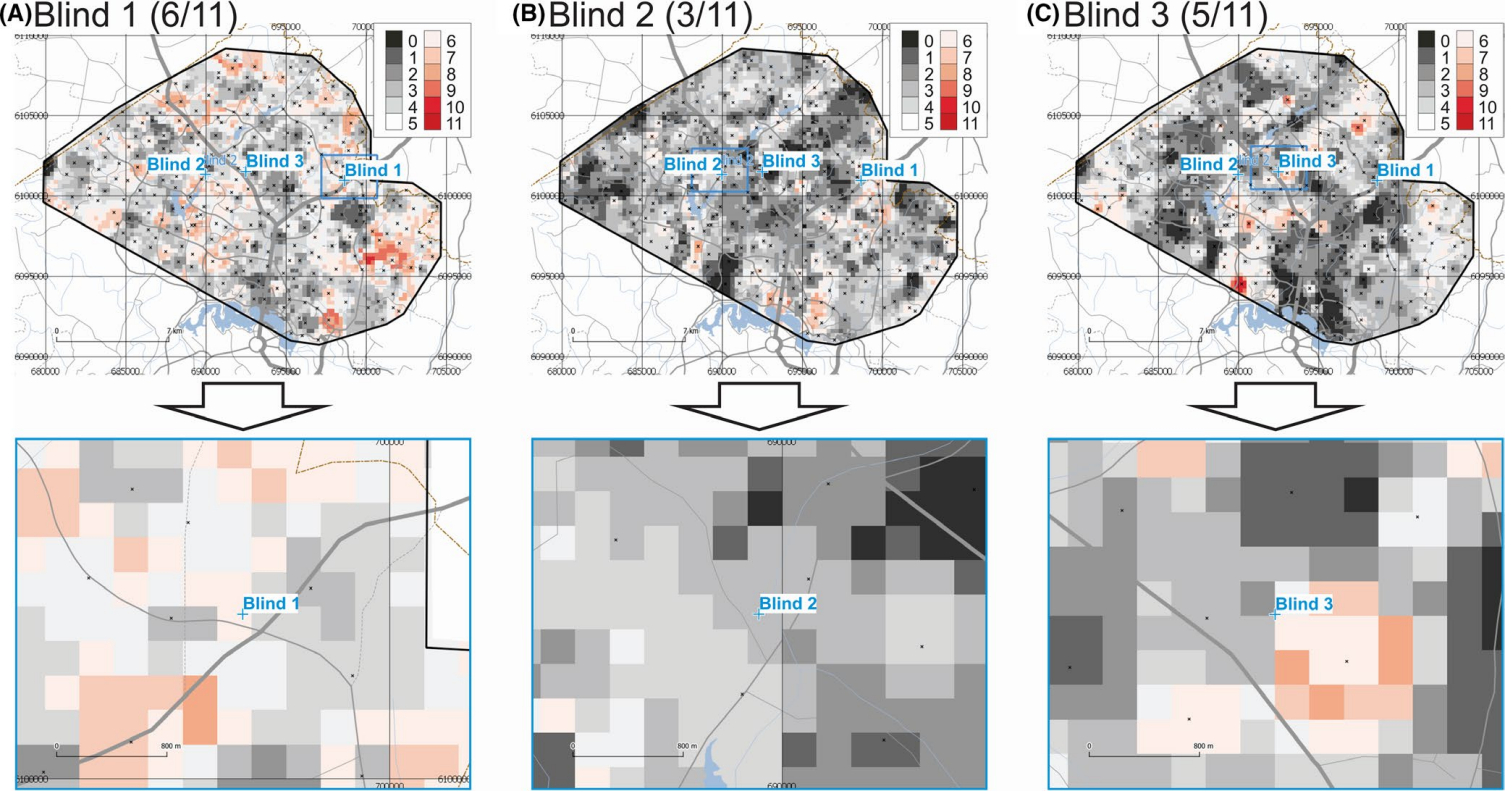
Provenance prediction maps for unknown samples Blind 1 (A), Blind 2 (B), and Blind 3 (C) based on XRF parameters (overview on top, detail below). Each raster cell gets a score of 1 if it satisfies the Search Range for any of these parameters, and 0 if not. Maximum theoretical score = 11. Geospatial data from ACT Government or Australian Government, unless otherwise indicated [Color figure can be viewed at wileyonlinelibrary.com]

The maps of provenance prediction for samples Blind 1, Blind 2, and Blind 3 based on 11 compositional XRF parameters are shown in Figure 4. Results indicate that for these three blind samples, 6 of 11, 3 of 11, and 5 of 11 parameters match the Search Ranges for Blind 1, Blind 2, and Blind 3, respectively. If the 8 first PCs are included in the analysis (not shown), the match rates for these three blind samples change to 10 of 19, 6 of 19, and 5 of 19 for Blind 1, Blind 2, and Blind 3, respectively.

**FIGURE 5:**
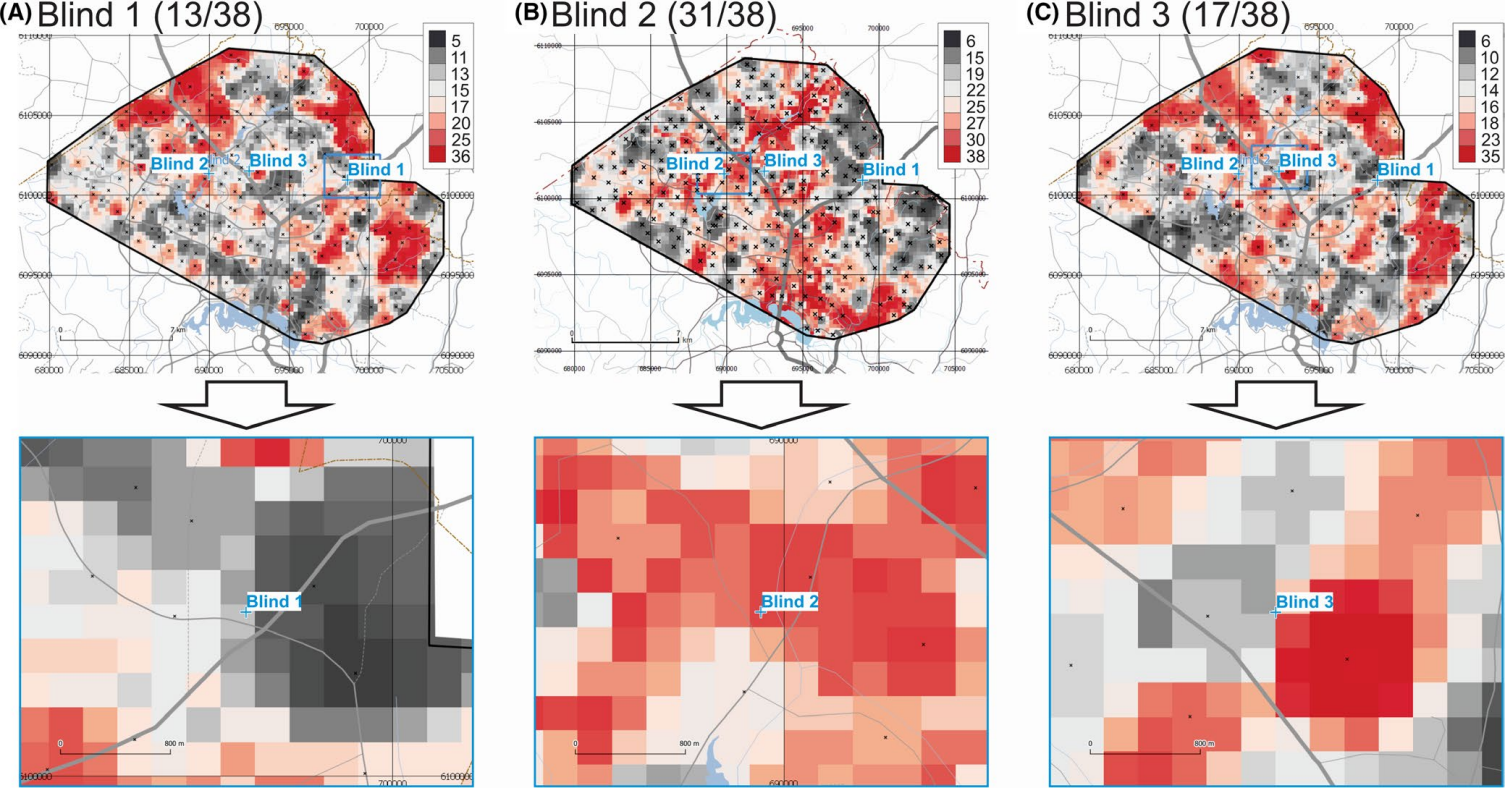
Provenance prediction maps for unknown samples Blind 1 (A), Blind 2 (B), and Blind 3 (C) based on Total ICP‐MS parameters (overview on top, detail below). Each raster cell gets a score of 1 if it satisfies the Search Range for any of these parameters, and 0 if not. Maximum theoretical score = 38. Color ramps are in quantile classes. Geospatial data from ACT Government or Australian Government, unless otherwise indicated [Color figure can be viewed at wileyonlinelibrary.com]

The maps of provenance prediction for samples Blind 1, Blind 2, and Blind 3 based on 38 compositional Total ICP‐MS parameters are shown in Figure 5. Results indicate that for these three blind samples, 13 of 38, 31 of 38, and 17 of 38 parameters match the Search Ranges for Blind 1, Blind 2, and Blind 3, respectively. If the 8 first PCs are included in the analysis (not shown), the match rates for these three blind samples change to 17 of 46, 39 of 46, and 29 of 46 for Blind 1, Blind 2, and Blind 3, respectively.

**FIGURE 6:**
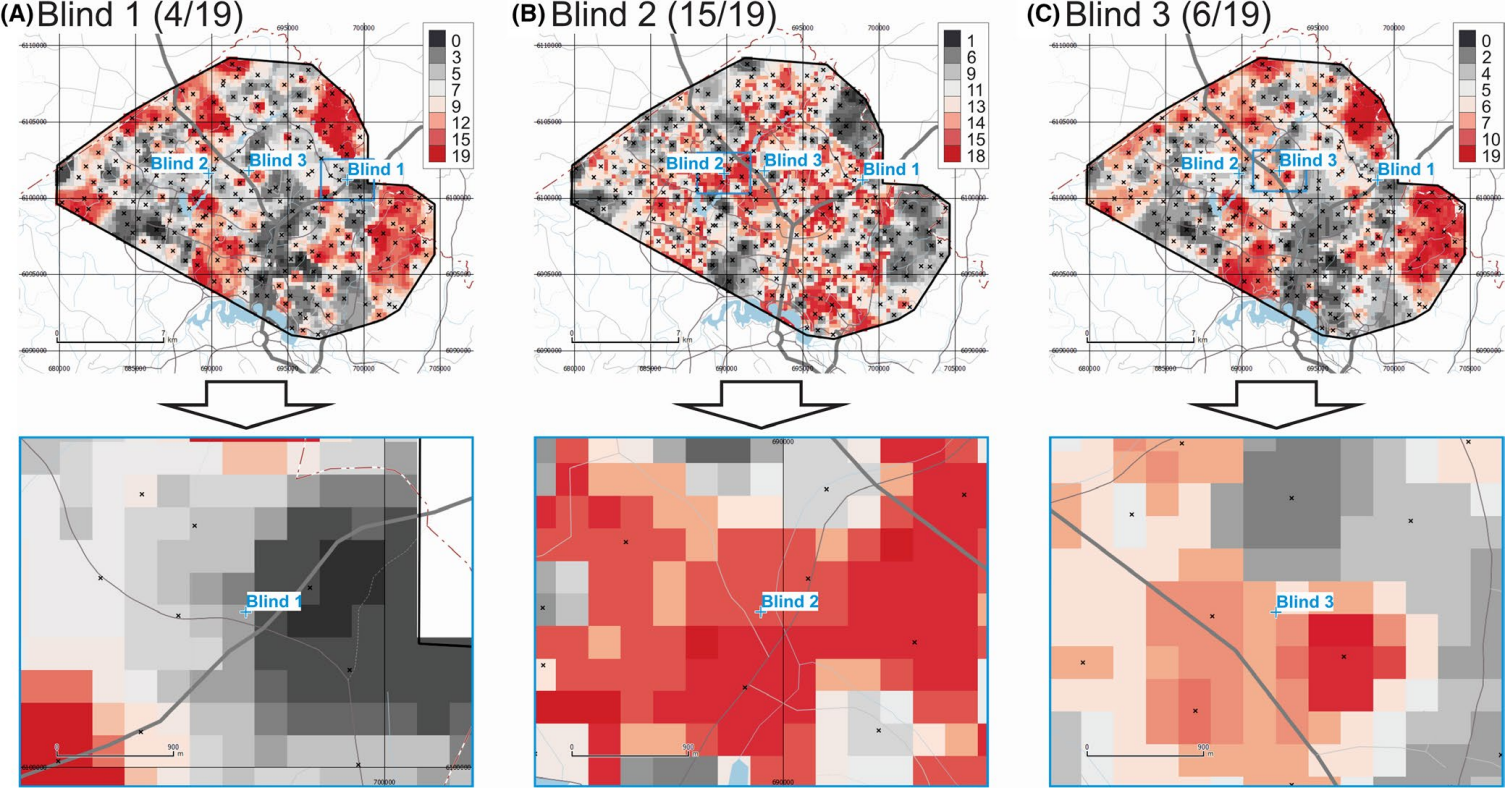
Provenance prediction maps for unknown samples Blind 1 (A), Blind 2 (B), and Blind 3 (C) based on AR ICP‐MS parameters (overview on top, detail below). Each raster cell gets a score of 1 if it satisfies the Search Range for any of these parameters, and 0 if not. Maximum theoretical score = 19. Color ramps are in quantile classes. Geospatial data from ACT Government or Australian Government, unless otherwise indicated [Color figure can be viewed at wileyonlinelibrary.com]

The maps of provenance prediction for samples Blind 1, Blind 2, and Blind 3 based on 19 compositional AR ICP‐MS parameters are shown in Figure 6. Results indicate that for these three blind samples, 4 of 19, 15 of 19, and 6 of 19 parameters match the Search Ranges for Blind 1, Blind 2, and Blind 3, respectively. If the 8 first PCs are included in the analysis (not shown), the match rates for these three blind samples change to 9 of 27, 20 of 27, and 10 of 27 for Blind 1, Blind 2, and Blind 3, respectively.

**FIGURE 7:**
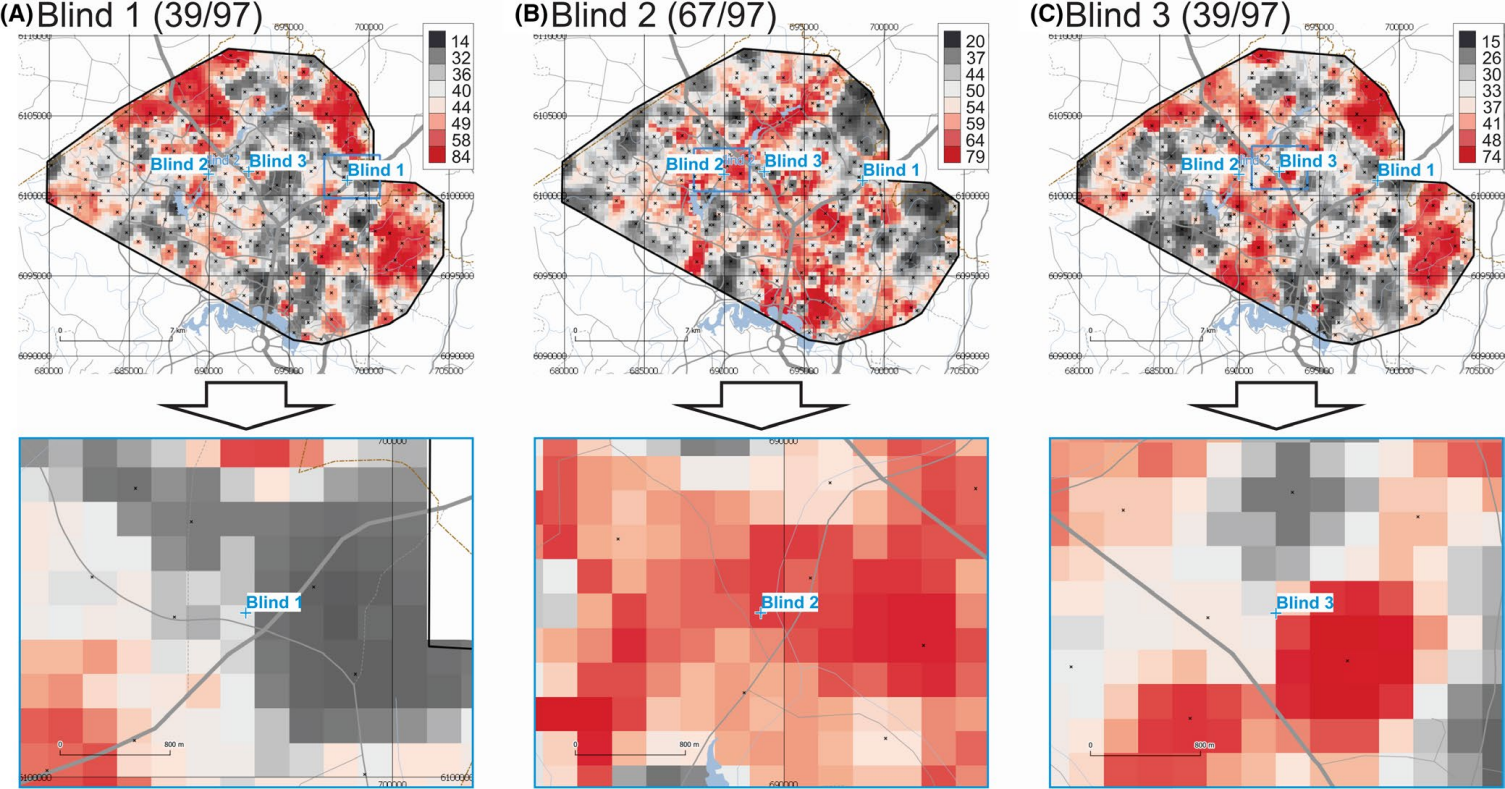
Provenance prediction maps for unknown samples Blind 1 (A), Blind 2 (B), and Blind 3 (C) based on All FTIR, MS, XRF, Tot, and AR parameters and their PCs (overview on top, detail below). Each raster cell gets a score of 1 if it satisfies the Search Range for any of these parameters, and 0 if not. Maximum theoretical score = 97. Color ramps are in quantile classes. Geospatial data from ACT Government or Australian Government, unless otherwise indicated [Color figure can be viewed at wileyonlinelibrary.com]

The maps of provenance prediction for samples Blind 1, Blind 2, and Blind 3 based on the 97 combined FTIR, MS, XRF, Tot, and AR ICP‐MS parameters (including PCs) are shown in Figure 7. Results indicate that for these three blind samples, 39 of 97, 67 of 97, and 39 of 97 parameters match the Search Ranges for Blind 1, Blind 2, and Blind 3, respectively. If the 27 PCs are removed from the analysis (not shown), the match rates for these three blind samples change to 26 of 70, 51 of 70, and 31 of 70 for Blind 1, Blind 2, and Blind 3, respectively.

### 3.2 Performance assessment

The performance statistics of the sequential method of provenancing soil samples are summarized in Table 5. Two performance indicators are calculated. First “precision” (Prc) is defined as the ratio of cells in a grid that have scores equivalent to, or lower than, the score of the cell containing the Blind (evidentiary) sample over the total number of cells. If Prc is 99%, only 1% of cells are identified as provenance matches, a highly precise result. Thus for instance, Blind 1 for FTIR +MS (5 variables) has a score of 3; there are 3634 cells of the total 4638 cells of the FTIR +MS grid that has a score of 3 or less (0, 1, or 2), giving Prc = 3634/4638 or 78.4%. Second “accuracy” (Acc) is defined as the ratio of the score for the cell containing the Blind (evidentiary) sample in a particular provenancing grid over the (actual) maximum score obtained at any cell within the grid. If Acc is 100%, all variables making up the provenance grid correctly identify the cell containing the evidentiary sample as a match, a highly accurate result. Thus for instance, Blind 1 for the FTIR +MS grid (maximum recorded score of 5) has a score of 3, giving Acc = 3/5 or 60%.

**TABLE 5:** Provenancing performance statistics for the sequential multivariate method for unknown samples Blind 1, Blind 2, and Blind 3 for all analytical methods, with and without principal components (PCs) included

Method	With PCs		Without PCs	
	Prc (%)	Acc (%)	Prc (%)	Acc (%)
FTIR + MS				
Blind 1	78.4	60.0	51.9	50.0
Blind 2	71.5	40.0	99.8	50.0
Blind 3	92.2	60.0	62.7	50.0
XRF				
Blind 1	93.6	66.7	86.6	60.0
Blind 2	73.0	42.9	64.8	33.3
Blind 3	64.1	33.3	88.7	50.0
AR				
Blind 1	35.9	34.6	25.6	21.1
Blind 2	92.3	80.0	92.1	83.3
Blind 3	66.8	38.5	63.3	31.6
Tot				
Blind 1	28.4	39.5	30.1	36.1
Blind 2	94.0	86.7	91.0	81.6
Blind 3	65.0	51.2	66.5	48.6
ALL				
Blind 1	40.4	46.4	30.6	40.0
Blind 2	93.1	84.8	90.3	78.5
Blind 3	65.7	52.7	70.7	50.0

Methods are as follows: Fourier transform infrared (FTIR), magnetic susceptibility (MS), X‐ray fluorescence (XRF), and aqua regia (AR) and total (Tot) inductively coupled plasma‐mass spectrometry; ALL represents all the above methods combined. Precision (Prc) is defined as the ratio of cells in a grid that have scores equivalent to, or lower than, the score of the cell containing the Blind (evidentiary) sample over the total number of cells. Accuracy (Acc) is defined as the ratio of the score for the cell containing the Blind (evidentiary) sample over the (actual) maximum score obtained at any cell within the grid. Prc and Acc reported in %. See text for details.

Each Blind sample behaves slightly differently in terms of provenancing performance (Table 5). The most precise and accurate results for Blind 1 were obtained for the XRF with PCs method (Prc = 93.6%; Acc = 66.7%). The next highest Prc came from XRF without PCs (86.6%), while the next highest Acc was obtained for both XRF without PCs and FTIR + MS with PCs (60%).

For Blind 2, the most precise results came from FTIR + MS without PCs (Prc = 99.8%), while the most accurate results were obtained for Total ICP‐MS with PCs (Acc = 86.7%). The next highest Prc came from Total ICP‐MS with PCs (94%), while the next highest Acc was obtained for ALL variables with PCs (84.8%).

The most precise and accurate results for Blind 3 were obtained for FTIR + MS with PCs (Prc = 92.2%; Acc = 60%). The next highest Prc came from XRF without PCs (88.7%), while the next highest Acc was obtained for ALL variables with PCs (52.7%) then Total ICP‐MS with PCs (51.2%).

Considering average rather than maximum (or next highest) precision and accuracy, provenancing performance improved from Blind 1 (50.1% and 45.4%), to Blind 3 (70.6% and 46.6%), to Blind 2 (86.2% and 66.1%). The authors believe that the poorer results for Blind 1 are due to the fact that this sample was collected in a non‐representative location for that grid cell (see above).

In terms of the best‐suited analytical method for deterministic provenancing across all Blind samples, FTIR + MS without PCs has the highest precision of any method (Prc = 99.8% for Blind 2), followed by Total ICP‐MS with PCs (94.0% for Blind 2), while Total ICP‐MS with PCs has the highest accuracy of any method (Acc = 86.7% for Blind 3), followed by ALL variables with PCs (84.8% for Blind 2) then AR ICP‐MS without PCs (83.3% for Blind 2).

Across all three Blind samples, FTIR + MS with PCs has the highest average precision (80.7%), closely followed by XRF without PCs (80.0%) then XRF with PCs (76.9%); ALL methods with PCs have the highest average accuracy (61.3%), followed by Total ICP‐MS with PCs (59.1%) then ALL methods without PCs (56.2%).

Inclusion of principal components (PCs) in the provenancing workflow provides a marginal advantage in terms of provenancing performance (Table 5). For all Blind samples confounded, both average Prc and Acc are higher with PCs (70.3% and 54.5%) than without (67.6% and 50.9%). From those figures, one can also observe that of the two performance metrics, Prc (ranges from 28.4% to 94.0% with PCs, and from 25.6% to 99.8% without PCs) tends to have higher values than Acc (ranges from 33.3% to 86.7% with PCs, and from 21.1% to 83.3% without PCs).

The performance of individual analytes, such as specific major oxides or trace elements, can be deduced from the sequential multivariate approach presented here. The following analytes were successful at matching all three Blind samples’ Search Ranges and can thus be put forward as the most effective soil provenancing indicators in the present study: Ba, Cu, Ge, Nb, Pb, Ta, and V by total ICP‐MS; Pb and Zn by *aqua regia* ICP‐MS; and two PCs for each of these analytical methods. However, seeing that analytical methods such as XRF or ICP‐MS are commonly available as packages of analytes rather than oxide‐by‐oxide or element‐by‐element analyses, realistically, comprehensive analyte packages are probably the most practical and cost‐effective requests to submit to institutional or commercial laboratories.

### 3.3 Sensitivity analysis

The sequential multivariate soil provenancing method developed here suggests a number steps to take for identifying regions within a search area (i.e., cells within a raster) that are more likely to contain the source of an evidentiary (blind) sample being provenanced. In this section, we test a number of variations on the previously described workflow to identify how sensitive the results are to parameterization choices. In particular, we measure the effect on the performance metrics Prc and Acc of (i) using an IDW algorithm with power of 2 (instead of 3) for the interpolation step, (ii) shifting the origin of the interpolation raster grids by 125 m to the west and south, (iii) using raster grid cells of 500 × 500 m (instead of 250 × 250 m), and (iv) applying an uncertainty multiplier of 6 (instead of 3) in calculating and propagating uncertainty (Equation 1). Table 6 shows the impact of these scenarios relative to the base scenario for XRF and Total ICP‐MS analyses.

**TABLE 6:** Sensitivity analysis of provenancing performance statistics for the sequential multivariate method for unknown samples Blind 1, Blind 2, and Blind 3 for X‐ray fluorescence (XRF) and total (Tot) inductively coupled plasma‐mass spectrometry analytical methods, with and without principal components (PCs) included

Method	With PCs		Without PCs		Scenario
	Prc (%)	Acc (%)	Prc (%)	Acc (%)	
XRF					
Blind 1	93.6	66.7	86.6	60.0	Sc 0
	95.4	88.9	95.3	80.0	Sc 1
	92.8	62.5	85.8	60.0	Sc 2
	99.6	88.9	98.8	90.9	Sc 3
	90.3	78.9	87.8	81.8	Sc 4
Blind 2	73.0	42.9	64.8	33.3	Sc 0
	66.0	70.6	65.7	44.4	Sc 1
	54.6	35.7	62.1	33.3	Sc 2
	73.1	70.6	79.3	72.7	Sc 3
	52.3	55.6	59.5	54.5	Sc 4
Blind 3	64.1	33.3	88.7	50.0	Sc 0
	66.3	66.7	66.0	40.0	Sc 1
	26.0	20.0	58.1	30.0	Sc 2
	23.3	38.9	48.6	54.5	Sc 3
	48.4	47.4	63.3	54.5	Sc 4
Tot					
Blind 1	28.4	39.5	30.1	36.1	Sc 0
	24.6	51.1	24.5	40.5	Sc 1
	7.2	30.2	0.8	24.3	Sc 2
	7.6	52.2	9.9	52.6	Sc 3
	14.8	63.0	15.6	60.5	Sc 4
Blind 2	94.0	86.7	91.0	81.6	Sc 0
	77.8	84.8	77.8	81.6	Sc 1
	76.7	75.6	77.6	75.7	Sc 2
	89.4	95.7	83.4	94.7	Sc 3
	94.2	97.8	92.8	97.4	Sc 4
Blind 3	65.0	51.2	66.5	48.6	Sc 0
	67.5	60.0	67.4	51.4	Sc 1
	51.5	46.3	52.5	42.9	Sc 2
	45.9	65.2	46.5	65.8	Sc 3
	82.4	87.0	83.9	86.8	Sc 4

The reference scenario (Sc 0) is the base case developed herein (IDW power 3; grid origin 679750,6090750; cell size 250 m x 250 m; and uncertainty multiplier 3). Variations modifying one of these parameters at a time are Sc 1 (IDW power 2), Sc 2 (grid origin 679625,6090625), Sc 3 (cell size 500 m x 500 m), and Sc 4 (uncertainty multiplier 6). Precision (Prc) and accuracy (Acc) reported in %. See text for details.

The sensitivity analysis (Table 6) reveals that performance metrics can vary by up to ±~40% relative to the reference scenario for Blinds 1, 2, and 3 combined and that Prc tends to deteriorate (−41% to +17%; i.e., negative bias) when parameters are changed, whereas Acc tends to improve (−20% to +39%; i.e., positive bias). Median changes in Prc relative to the base scenario are −10% and −7% with and without PCs, respectively. Median changes in Acc relative to the base scenario are +12% and +8% with and without PCs, respectively. The dependency of provenancing performance on parameter choices is relatively significant: performance across all three Blind samples and five scenarios averages 61.5% and 64.4% for Prc when PCs are and are not included, respectively, and 61.8% and 59.4% for Acc when PCs are and are not included, respectively. Therefore, we recommend that values of 60% be used for both Prc and Acc as minimum thresholds for accepting a provenance prediction. On this basis, Table 5 clearly shows that provenancing of Blind 1 largely failed (4 out of 20 performance metrics ≥60%), most likely because of the uncharacteristic choice of location of this blind sample as discussed above, whereas provenancing of Blind 3 (10 out of 20) and especially Blind 2 (16 out of 20) was (more) successful.

### 3.4 Limitations and future research

The present study focussed specifically on data analysis workflows for the provenancing of soil trace evidence. It did not address the (acknowledged) issues of (i) sample size available for analysis in a geochemical survey situation *vs* a crime scene forensic casework; (ii) soil transfer and persistence from the crime scene to the point where soil is sampled for forensic assessment; (iii) the potential for a questioned soil sample from an urban/suburban environment being impacted by human activity (e.g., transported soil for landscaping or engineering purpose); and (iv) the choice of interpolation method to predict the values of a soil property between survey grid points. The latter point has been the focus of investigations in the past (e.g., [39, 40, 41, 42]), though perhaps not specifically with a forensic application in mind. Other limitations to this provenancing approach, such as contamination, are common to all forensic traces, for example, fingerprinting, biological tissues, fibers, and not specific to soil provenancing; they are of course an important concern and need to be managed by appropriate protocols.

Future research could thus include expanding the present investigation to include (i) micro‐analysis techniques, and (ii) quantitative mineralogical and geochemical assessment of soil transfer and persistence (e.g., as footsteps are taken with dirty boots, a car is driven with muddied tires, or a shovel is subjected to drying and shaking to simulate transport in a vehicle).

Despite the acknowledged limitations to the empirical soil provenancing approach developed herein and the recognition that additional research is recommended, it is concluded that empirical soil provenancing based on soil mineralogical and geochemical surveys can play an important role in forensic and intelligence applications.

## 4 SUMMARY AND CONCLUSIONS

A sequential multivariate method of soil provenancing was applied to a high‐density (1 sample per km^2^) soil geochemical survey around North Canberra, southeastern Australia. In this survey, 268 air‐dried topsoil samples (0–5 cm) were analyzed for mineralogical and geochemical properties (Fourier transform infrared (FTIR) spectroscopy, magnetic susceptibility (MS), and geochemical composition by X‐ray fluorescence (XRF) for total major oxides, and inductively coupled plasma‐mass spectrometry (ICP‐MS) for total trace elements as well as for *aqua regia*‐soluble trace elements). Quality control measures, including the analysis of several sample triplicates, internal project standards, and certified reference materials, were applied. Appropriate compositional data and multivariate statistical analyses were carried out, including imputation of censored values, centered log‐ratio transformation, and calculation of principal components (PCs). Uncertainty was explicitly quantified and propagated through all computations. Three blind samples, whose locations were unknown to the principal investigator, were collected by an Australian Federal Police forensic scientist to simulate forensic soil evidence to be provenanced by the method. These Blind samples were analyzed by the same techniques and their Target Values (TVs) and Search Ranges (SRs) determined, where SR =TV ± total uncertainty.

The multivariate sequential provenancing method consists of preparing an interpolated soil property raster for each reported mineralogical/geochemical variable. This was done here using the common inverse distance weighting (power of 3) interpolation method using a grid cell size of 250 × 250 m. Interpolation uncertainty was determined for every soil property raster. For any measured variable, each grid cell receives a score of 1 where its interpolated value (± uncertainty) overlaps with the uncertainty envelope of the evidentiary soil sample, or 0 otherwise. The score grids are calculated sequentially for all variables and added up to produce “heat maps” showing the pixels where most properties from the survey match (within uncertainty) those of the evidentiary sample.

The three blind samples showed a range of performance against the various analytical methods, with Blind 1 performing on average the worst and Blind 2 the best. This indicates that an evidentiary sample may not always be representative of a sample taken for geochemical mapping purposes. Thus, natural soil heterogeneity is potentially a challenge requiring further research in forensic provenancing.

The most precise analytical methods for soil provenancing identified in this study are FTIR + MS with PCs (average precision 80.7%), closely followed by XRF without PCs (80.0%) then XRF with PCs (76.9%). The most accurate analytical methods are ALL methods with PCs (average accuracy 61.3%), followed by Total ICP‐MS with PCs (59.1%) then ALL methods without PCs (56.2%). We conclude that (i) empirical soil provenancing should prioritize FTIR, MS, and XRF analysis, followed by Total ICP‐MS and lastly AR ICP‐MS; (ii) combining mineralogical information (e.g., FTIR or MS here, but potentially also X‐ray diffraction, etc.) with geochemistry significantly enhanced the performance of soil provenance analysis; (iii) having access to as comprehensive an analytical suite as possible is advantageous as shown by the performance of the ALL methods category; and (iv) inclusion of PCs in the provenancing workflow provides a marginal advantage in terms of provenancing performance compared to not considering PCs. In a companion paper, we will investigate a simultaneous, rather than sequential, empirical soil provenancing method.

## Supporting information

Supplementary Material
