# Supplementary material for: Forensic soil provenancing in an urban/suburban setting: A sequential multivariate approach
Source: J Forensic Sci. 2021 May 6;66(5):1679–96. doi: 10.1111/1556-4029.14727 (PMC8453720; doi:10.1111/1556-4029.14727)
Supplement: Supplementary file 1 — Supplementary Material [file JFO-66-1679-s001.pdf]

Forensic soil provenancing in an urban/suburban setting: A sequential multivariate approach

Patrice de Caritat[1,2,3, †,*], Brenda Woods[1,3], Timothy Simpson[1], Christopher Nichols[3,4] , Lissy Hoogenboom[3], Adriana Ilheo[3,5], Michael G. Aberle[3], Julian Hoogewerff[3]

[1]Australian Federal Police, GPO Box 401, Canberra ACT 2601, Australia

[2]Permanent Address: Geoscience Australia, GPO Box 378, Canberra ACT 2601, Australia

[3]National Centre for Forensic Studies, University of Canberra, Bruce ACT 2601, Australia

[4]Current Address: UQ Centre for Natural Gas, The University of Queensland, St Lucia QLD 4072, Australia

[5]Current Address: Australian Federal Police, GPO Box 401, Canberra ACT 2601, Australia

[†]ORCID: 0000-0002-4185-9124

[*]Corresponding author: Patrice de Caritat, Geoscience Australia. Phone: +612 6249 9378. Email: Patrice.deCaritat@ga.gov.au

SUPPLEMENTARY MATERIAL – APPENDIX S1

1. METHODS

1.1. Sample Collection, Preparation and Analysis

This urban/suburban soil geochemistry project was undertaken to provide a field-based demonstration of the empirical forensic soil provenancing methodology. 324 topsoil samples (including field triplicates at 28 of the sites) were collected from 268 locations across north Canberra (ACT) in winter and spring of 2017 and summer 2017-18 at a nominal density of ~1 sample per 1 km^2. Each sample was a ~1 kg composite of five subsamples taken at the corners and centre of a 1 m x 1 m patch of soil, with each subsample being ~15 cm x 15 cm x 5 cm deep after removal of surface vegetation. GPS coordinates and site descriptions were recorded at each location and digital photographs were taken. Urban and suburban public parks and reserves, as well as private grazing pasture fields were sampled with an emphasis on avoiding obviously disturbed, contaminated or engineered soils. In addition to the standard survey samples, three 'blind' samples were collected for the purpose of validating the provenancing methodology (Fig. S1). Full details of the method are given in Nichols (43).

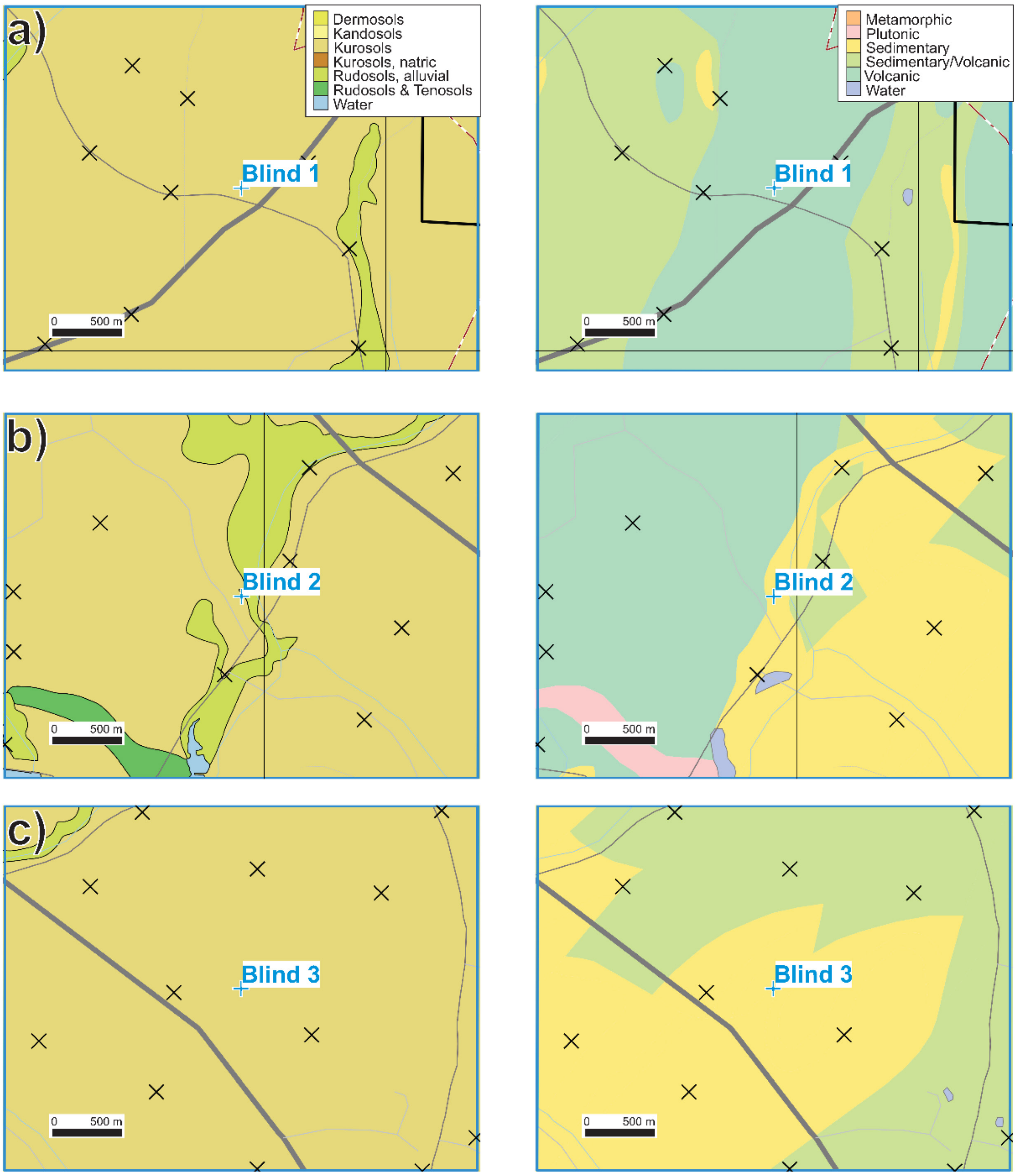

FIGURE S1. Detailed locations of Blind 1 (a), Blind 2 (b), and Blind 3 (c) samples (plusses) with surrounding survey samples (crosses) for the North Canberra, Australian Capital Territory (ACT), soil geochemical survey overlain on Australian Soil Classification (ASC) soil orders (left), and high-order lithology types (geology) (right). Principal, main, and secondary roads are shown as thick, thin, and dashed gray lines, respectively. Water bodies and drainage are shown in blue. ACT border is shown as brown dash-dotted line. Geospatial data from ACT Government or Australian Government, unless otherwise indicated

In the laboratory the soil samples were dried at 40 °C for 48 h in a Thermoline Australia® D100 fan-forced dehydrating oven, then sieved to <75 µm using a nylon mesh Flexistack® sieve and sample collector. Analytical protocols applied are described below.

Attenuated Total Reflectance (ATR)-Fourier Transform InfraRed (FTIR) spectroscopy was performed on a Thermo Scientific® Nicolet iS50 FT-IR spectrometer equipped with an iS50 ATR detector and pressure-gauged anvil at the AFP Forensics Laboratory (Majura, ACT). A calibration using a Thermo Scientific serialized 3 mm thick polystyrene standard was performed at the beginning of each data acquisition session. Approximately 100 mg of sample were placed in a custom-made aluminium stage to ensure the same volume of material (4 mm diameter x 1 mm thickness) was being analysed every time. Measurement settings were 64 scans, resolution 4 cm^{-1}, range 400 to 4000 cm^{-1} (mid- and far-IR spectra), at laboratory temperature. A background reading was taken under the same conditions before each sample measurement. In the interest of time, most samples were run only once, except for approximately 5% of them, which were run at least in triplicate. The OMNIC software (v. 9.8.286) was used to acquire, display and store the data, the latter in proprietary .spa and open source .csv formats. Absorbance against wavenumber was recorded.

Magnetic Susceptibility (MS) of the soil samples was measured using a Bartington® MS2B dual frequency sensor coupled to a Bartington® MS3 meter at the Black Mountain Paleomagnetic Laboratory of ANU's Research School for Earth Sciences. The MS2B/MS3 system had a resolution of 2 x 10^{-6} SI and a precision of 1%. Measurements were conducted on cubic 20 mm x 20 mm x 20 mm (8 cm^3) clear polycarbonate sample holders (cells) manufactured by AGICO®. The net mass of each sample was measured and recorded. Each MS measurement consisted of a blank/sample/blank recording cycle, each of 1 s duration. Each sample was measured at least in triplicate. Every 20 samples, an empty cell as well as two samples (ICJ08291 and GCG06262 of low and high MS, respectively) were remeasured for quality control. In addition a Kappabridge® calibration standard of known volume and specific MS (80.28 10^{-3} SI) was run several times over the data acquisition period. Bartington's Bartsoft software (v. 4.2.1.1) was used to acquire, display and store the data, the latter in proprietary .bid and open source .txt formats. Volume-specific MS (K or 'kappa') was recorded in SI units at low frequency (lf; 465 Hz) and again at high frequency (hf; 4650 Hz), from which mass-specific MS (X or 'chi', in units of 10^{-6} m^3/kg) and percent frequency-dependent MS (Xfd, in units of %) were calculated as:

$$X_{lf} = K_{lf} \times \frac{Volume\ (m^3)}{Mass\ (kg)}, \text{and } X_{hf} = K_{hf} \times \frac{Volume\ (m^3)}{Mass\ (kg)} \quad \text{(Eqn 3)}$$

$$X_{fd}\,(\%) = 100 \times \frac{|X_{hf} - X_{lf}|}{X_{lf}} \quad \text{(Eqn 4)}$$

X-ray fluorescence (XRF) bead preparation, fusion and analysis were performed at the GA Inorganic Laboratory in Canberra. A mass of 1.000 (± 0.001) g of dried sample was accurately weighed and mixed with 6.000 (± 0.001) g of flux. For these silica-rich samples, the recommended LM100 flux (100% lithium metaborate, $LiBO_2$) was used. The resulting mixtures were fused under agitation at 1050 °C in platinum crucibles placed in a furnace. Once cooled, the resulting glass beads were analysed on a Bruker® S8Tiger wavelength dispersive XRF analyser equipped with an autosampler. The concentrations of 10 major oxides (SiO_2, TiO_2, Al_2O_3, Fe_2O_3, MnO, MgO, CaO, Na_2O, K_2O, P_2O_5) were determined and reported in weight percent (wt%) by this method. Practical lower limits of detection obtained by this method are given in Table S1. Full details of the method are given in (44-45).

TABLE S1. Practical lower limits of detection (*LLD*) obtained for each analyte, as determined by X-ray fluorescence (_XRF), or by inductively coupled plasma-mass spectrometry after aqua regia (_AR) and total (_Tot) digestion. Units are: weight percent (wt%) or parts per million (ppm; equivalent to mg/kg). See text for details

**Variable**	*Units*	*LLD*	**Variable**	*Units*	*LLD*
**SiO2_XRF**	wt%	0.01	**As_Tot**	ppm	0.18
**TiO2_XRF**	wt%	0.01	**Ba_Tot**	ppm	0.42
**Al2O3_XRF**	wt%	0.01	**Bi_Tot**	ppm	0.06
**Fe2O3_XRF**	wt%	0.01	**Ce_Tot**	ppm	0.03
**MnO_XRF**	wt%	0.01	**Co_Tot**	ppm	0.15
**MgO_XRF**	wt%	0.05	**Cr_Tot**	ppm	1.05
**CaO_XRF**	wt%	0.01	**Cs_Tot**	ppm	0.06
**Na2O_XRF**	wt%	0.01	**Cu_Tot**	ppm	0.45
**K2O_XRF**	wt%	0.01	**Dy_Tot**	ppm	0.15
**P2O5_XRF**	wt%	0.01	**Er_Tot**	ppm	0.03
**LOI_XRF**	wt%	0.01	**Eu_Tot**	ppm	0.03
**Ba_AR**	ppm	0.03	**Ga_Tot**	ppm	0.09
**Be_AR**	ppm	0.01	**Gd_Tot**	ppm	0.03
**Ce_AR**	ppm	0.005	**Ge_Tot**	ppm	0.09
**Co_AR**	ppm	0.02	**Hf_Tot**	ppm	0.06
**Cs_AR**	ppm	0.30	**Ho_Tot**	ppm	0.03
**Dy_AR**	ppm	0.002	**La_Tot**	ppm	0.06
**Er_AR**	ppm	0.004	**Lu_Tot**	ppm	0.09
**Eu_AR**	ppm	0.001	**Nb_Tot**	ppm	0.06
**Fe_AR**	ppm	100	**Nd_Tot**	ppm	0.06
**La_AR**	ppm	0.002	**Ni_Tot**	ppm	2.40
**Mn_AR**	ppm	0.20	**Pb_Tot**	ppm	0.15
**Nd_AR**	ppm	0.002	**Pr_Tot**	ppm	0.03
**Ni_AR**	ppm	0.04	**Rb_Tot**	ppm	0.30
**Pb_AR**	ppm	0.20	**Sc_Tot**	ppm	0.15
**Rb_AR**	ppm	0.10	**Sm_Tot**	ppm	0.30
**Sr_AR**	ppm	0.03	**Sn_Tot**	ppm	0.15
**Th_AR**	ppm	0.50	**Sr_Tot**	ppm	0.30
**Tm_AR**	ppm	0.003	**Ta_Tot**	ppm	0.03
**Zn_AR**	ppm	15	**Tb_Tot**	ppm	0.03
			**Th_Tot**	ppm	0.03
			**U_Tot**	ppm	0.03
			**V_Tot**	ppm	0.30
			**W_Tot**	ppm	0.12
			**Y_Tot**	ppm	0.06
			**Yb_Tot**	ppm	0.06
			**Zn_Tot**	ppm	1.20
			**Zr_Tot**	ppm	0.15

Loss on ignition (LOI) was measured at the GA Inorganic Laboratory in Canberra. This analyte is often bundled together with the XRF results because when considered together the major element oxides plus LOI, all expressed in wt%, should amount to close to 100 wt%, i.e., a full total (closed and complete) analysis. LOI reflects 'combined' water (soil moisture plus mineral-bound water), CO_2 from carbonates and organic matter (and potentially other volatiles) contained in the soil. To measure LOI a sample mass of approximately but accurately weighed 1000 mg was added to a tared porcelain crucible, which was then placed in a furnace at 1000 °C. When ignition was completed, the crucible was allowed to cool down before reweighing and the LOI was calculated by difference and reported in wt%. Practical lower limits of detection obtained by this method is given in Table S1. Full details of the method are given in (46).

Total digestion ICP-MS was performed at the GA Inorganic Laboratory in Canberra, using an Agilent® 7500ce quadrupole inductively coupled plasma-mass spectrometer (ICP-MS) equipped with an octopole reaction cell and an autosampler. Fragments of the glass beads prepared for XRF analysis (to an accurately measured mass of 0.1 g) were digested in 5 mL of an acid mixture consisting of 1:5:4 (vol) ultrahigh purity $HF:HNO_3:H_2O$ on a shaking platform for at least 4 h at room temperature (volumed to 100 mL with Milli-Q 'type 1' water). Internal standards, certified reference materials (CRMs), blanks, spiked samples and replicates were analysed for quality control and interference corrections applied. The total concentration of 38 elements (As, Ba, Bi, Ce, Co, Cr, Cs, Cu, Dy, Er, Eu, Ga, Gd, Ge, Hf, Ho, La, Lu, Nb, Nd, Ni, Pb, Pr, Rb, Sc, Sm, Sn, Sr, Ta, Tb, Th, U, V, W, Y, Yb, Zn, Zr) were determined and reported in mg/kg (= parts per million or ppm) for all samples. Practical lower limits of detection obtained by this method are given in Table S1. Full details of the method are given in (47-49).

Aqua regia (AR, 3:1 mol:mol $HCl:HNO_3$) digestion and analysis were performed at the UC laboratory, using a PerkinElmer® NexION 300D inductively coupled plasma-mass spectrometer (ICP-MS). A range of multi-element CRMs and external standards, as well as blanks and replicate samples, were included for quality control. A mass of 0.25 (± 0.05) g of sample was digested in 2.4 mL AR solution, first at room temperature for at least three days followed by 2 h on an 80 °C heat block. The concentrations of 19 AR-soluble elements (Ba, Be, Ce, Co, Cs, Dy, Er, Eu, Fe, La, Mn, Nd, Ni, Pb, Rb, Sr, Th, Tm, Zn) were determined and reported in mg/kg (= parts per million or ppm) for all samples. Practical lower limits of detection obtained by this method are given in Table S1. Full details of the method are given in (43).

1.2. Data Analysis

In terms of data analysis, the emphasis of this study was to apply multivariate statistics and geographical information systems approaches. In addition, geochemical data was treated in

accordance to compositional data analysis (CODA) principles, to ensure appropriate statistical analysis. The data analysis methods are described below.

All compositional data (XRF, AR ICP-MS and Total ICP-MS) were first checked for the presence of censored data (values below the lower limit of detection, LLD). Censored data are important to preserve in subsequent statistical analysis, and should not be discarded; it is indeed valuable to know that the concentration of an element was measured and found to be low, in fact so low that it falls within the noise of the measurement technique. In the present study, censored data were observed for Cs and Zn (AR) and for Bi and Ni (Tot) only. These values were replaced by imputed values obtained by the nearest neighbour methodology using the 'impRZilr' function, which employs an isometric log-ratio (ilr) transformation of the data, of the 'robCompositions' package (50) in the R statistical package (51).

The next step of compositional data preparation involved clr transformation. This is one of the most commonly applied transformations in CODA (52), and involves dividing each concentration value by the geometric mean of that sample (with all original variables expressed in the same concentration units) and taking the natural logarithm of that ratio. This has the advantage of removing the 'closure' problem of compositional data and rendering originally 'relative' data reported in proportional units (e.g., parts per millions) 'absolute' and reported in dimensionless units. The clr transformation for a vector x (observation) consisting of D elements (variables) is calculated as:

$$clr(x) = \left[ln\left(\frac{x_1}{g(x)}\right), ln\left(\frac{x_2}{g(x)}\right), \ \ldots, ln\left(\frac{x_D}{g(x)}\right)\right] \quad \text{(Eqn 5)}$$

where g(x) is the geometric mean of sample x. Campbell et al. (53) have discussed CODA aspects of geochemical data in a forensic context.

Principal component analysis (PCA) is a common multivariate statistical method that extracts independent (orthogonal) linear combinations of variables that explain the variance in a dataset. The first principal component (PC1) explains by itself the largest amount of variance, PC2 the next largest amount, and so forth. With only a few PCs it is thus possible to capture the vast majority of the variance (i.e., information) in a complex multivariate dataset. In this work, PCA was conducted on chemical data (XRF, Total ICP-MS, and AR ICP-MS) after clr-transformation of the variables using a Python script developed at Geoscience Australia (Philip Main, unpublished) that works in combination with ESRI's ArcGIS. The variances explained by the dominant PCs for each analytical method are given in Table S2.

TABLE S2. Proportions of the variance (*Var*) explained by each principal component (PC1, PC2, …), individually and cumulatively (*Cumul*; up to 0.97 shown). Methods are: Fourier Transform InfraRed (_FTIR), X-ray fluorescence (_XRF), and aqua regia (_AR) and total (_Tot) inductively coupled plasma-mass spectrometry. Units are: All PCs: dimensionless. See text for details

Variable	*Var*	*Cumul*	Variable	*Var*	*Cumul*
**PC1_FTIR**	0.89	0.89	**PC1_Tot**	0.27	0.27
**PC2_FTIR**	0.07	0.95	**PC2_Tot**	0.23	0.50
**PC3_FTIR**	0.02	0.97	**PC3_Tot**	0.09	0.59
**PC1_XRF**	0.29	0.29	**PC4_Tot**	0.06	0.65
**PC2_XRF**	0.19	0.47	**PC5_Tot**	0.05	0.70
**PC3_XRF**	0.15	0.63	**PC6_Tot**	0.04	0.75
**PC4_XRF**	0.12	0.75	**PC7_Tot**	0.04	0.78
**PC5_XRF**	0.09	0.84	**PC8_Tot**	0.03	0.81
**PC6_XRF**	0.05	0.89	**PC9_Tot**	0.02	0.83
**PC7_XRF**	0.04	0.93	**PC10_Tot**	0.02	0.85
**PC8_XRF**	0.03	0.96	**PC11_Tot**	0.02	0.87
**PC9_XRF**	0.02	0.98	**PC12_Tot**	0.02	0.89
**PC1_AR**	0.31	0.31	**PC13_Tot**	0.02	0.90
**PC2_AR**	0.17	0.48	**PC14_Tot**	0.01	0.92
**PC3_AR**	0.11	0.59	**PC15_Tot**	0.01	0.93
**PC4_AR**	0.09	0.69	**PC16_Tot**	0.01	0.94
**PC5_AR**	0.07	0.76	**PC17_Tot**	0.01	0.95
**PC6_AR**	0.06	0.81	**PC18_Tot**	0.01	0.95
**PC7_AR**	0.04	0.86	**PC19_Tot**	0.007	0.96
**PC8_AR**	0.03	0.89	**PC20_Tot**	0.006	0.97
**PC9_AR**	0.03	0.92	**PC21_Tot**	0.005	0.97
**PC10_AR**	0.02	0.94			
**PC11_AR**	0.02	0.96			
**PC12_AR**	0.02	0.97			

For the FTIR dataset, PCA was conducted using the R package 'ChemoSpec' (54), which is specifically designed for chemometric analysis of spectroscopic data. Of the original 7700 wavenumbers recorded for each analysis between 400 and 4000 cm^{-1}, the non-relevant interval 1800-2749 cm^{-1} was first truncated (using command removeFreq()), leaving 5500 wavenumbers (variables) to analyse and interpret. PCA was conducted after centring (using command c_pcaSpectra()). A sensitivity analysis comparing the various normalisation and scaling options available in the package concluded that the highest proportions of variance in the first three PCs was obtained when no normalisation and no scaling were applied; thus these options were applied here.

1.3. Spatial Analysis

Open access geographical information system software QGIS version 2.18 or 3.4 was used for all spatial analysis. Interpolation rasters were generated for all available variables from all the (known)

samples in the database using the inverse distance weighting (IDW) algorithm in QGIS. This method is underpinned by the concept of spatial dependency or autocorrelation, namely observations that are spatially close are more likely to be more similar than observations that are further apart. This generally applies to soils as noted in the introduction to the main article, though of course abrupt gradients or discontinuities can occur in nature. Other interpolation methods were tested, including nearest neighbours, spline and kriging, and IDW was found to be best-suited (smallest residuals).

Thus, the value of a soil property at an unsampled point (z_p) was calculated from the closest n sampled sites (z_i) using the IDW algorithm:

$$z_p = \left[\sum_{i=1}^{n} \left(z_i / d_i^p \right) \Big/ \sum_{i=1}^{n} \left(1 / d_i^p \right) \right] \quad \text{(Eqn 6)}$$

where n is the number of neighbouring samples taken into account (here 12, a commonly used value and the default in QGIS), d_i is the distance between the unsampled point and sampled site i, and p is the power or exponent. Values from the QC sites sampled in triplicate were averaged prior to this interpolation. The IDW interpolation applied here uses a power of 3, instead of the more commonly used (default) value of 2. This choice was made after testing a range of power values (1.5, 2, 2.5, and 3), with the smallest residuals (i.e., discrepancies between measured and modelled values) being obtained with a power of 3.

Raster cell size was set to a fairly coarse 250 m x 250 m. This choice was made to avoid over-interpreting the raster grids obtained from a dataset with a nominal spatial density of 1 site per km^2, yet allow sufficient smoothing of gradients to infer meaningful trends and patterns. It is consistent with the common raster data practice (55).

Extent of interpolation was set to Xmin = 679,750 m; Xmax = 704,750 m; Ymin = 6,090,750 m; Ymax = 6,109,250 m (Spatial Reference EPSG:28355, MGA Zone 55, GDA 1994 datum). This gave a basic rectangular raster of size 100 columns x 74 rows (7400 cells) for each variable. Batch IDW raster creation for all available variables was performed using the GRASS GIS7 'v.surf.idw' tool in QGIS.

The generated rasters were subsequently clipped to a polygon circumscribing the extent of the sampled area, using a polygon shapefile following the study area outline of Fig. 1. This clipping step avoided that subsequent data analysis and raster statistics incorporated cells that are not supported by (i.e., far removed from) data points. The clipped rasters contain 4638 cells each (calculated using the SAGA 2.3.2 'Zonal raster statistics' tool in QGIS) all registered to the exact same grid. Batch raster clipping for all generated rasters was performed using the SAGA 2.3.2 'Clip raster with polygon' tool in QGIS.

Raster calculations, such as the “AND” logical operator to sum raster scores cell by cell, were performed using QGIS inbuilt tools. Raster statistics, such as counts, min, median and max, were performed using SAGA 2.3.2 ‘Zonal raster statistics’ tool in QGIS. Residuals, which are the differences between the interpolated (modelled) values and the measured values at each sampled site, were obtained using the ‘Point sampling tool’ plugin (v. 0.4.2) developed by Borys Jurgiel in QGIS.

1.4. Quality Control and Uncertainty Analysis

Of the 268 sites selected for the north Canberra geochemical survey, 28 were randomly selected for the collection of triplicate samples to quantify site selection uncertainty. Analytical uncertainty was quantified by analysing a suite of NGSA (56) and GEMAS (57) samples and internal project standards (WRIS-2, GEMAS AP, GEMAS AR), one internal project standard (LBC1112), as well as certified reference materials (WG-1, TILL-1, TILL-2) and replicate (split) samples (lab duplicates) and replicate analyses (repeats). Measurement and interpolation standard deviations (SD_m and SD_i) are given for each analyte in Table S3. Relative standard deviations (RSDs) were calculated from SD_m (Table S3) and *Ave* (Table 1) for each analyte and were averaged by method to give 6.7% for MS, 5.7% for XRF, 5.9% for AR ICP-MS, and 5.8% for Tot ICP-MS.

Measurement, interpolation and combined uncertainties (U_m, U_i, U_c) calculated as per equation in the main paper (Uncertainty Analysis section) are given for each analyte in Table S3.

TABLE S3. Empirically derived measurement (*SDm*) and interpolation (*SDi*) standard deviations, and measurement (*Um*), interpolation (*Ui*) and combined (*Uc*) uncertainties for all variables. Methods are: Fourier Transform InfraRed (_FTIR), mass-specific (Xlf) and frequency-dependent in percent (Xfd_pc) Magnetic Susceptibility, X-ray fluorescence (_XRF), and aqua regia (_AR) and total (_Tot) inductively coupled plasma-mass spectrometry. Units are: All PCs: dimensionless; Xlf: 10^{-6} m^3/kg; Xfd_pc: %; XRF: wt%; AR and Tot: mg/kg (ppm). See text for details

Variable	*SDm*	*SDi*	*Um*	*Ui*	*Uc*	Variable	*SDm*	*SDi*	*Um*	*Ui*	*Uc*
**PC1_FTIR**	0.32	0.03	0.95	0.10	0.96	**As_Tot**	0.18	0.09	0.55	0.28	0.62
**PC2_FTIR**	0.02	0.01	0.05	0.04	0.06	**Ba_Tot**	12.8	2.15	38.3	6.44	38.8
**PC3_FTIR**	0.04	0.005	0.12	0.02	0.13	**Bi_Tot**	0.05	0.01	0.16	0.04	0.16
**Xlf**	0.10	0.04	0.29	0.12	0.31	**Ce_Tot**	1.63	0.55	4.88	1.64	5.14
**Xfd_pc**	0.20	0.05	0.60	0.15	0.61	**Co_Tot**	0.35	0.09	1.04	0.28	1.08
**SiO2_XRF**	0.34	0.11	1.03	0.32	1.08	**Cr_Tot**	1.75	0.26	5.25	0.77	5.31
**TiO2_XRF**	0.008	0.002	0.03	0.007	0.03	**Cs_Tot**	0.17	0.03	0.50	0.08	0.50
**Al2O3_XRF**	0.15	0.05	0.45	0.15	0.47	**Cu_Tot**	5.40	0.21	16.2	0.62	16.2
**Fe2O3_XRF**	0.15	0.02	0.44	0.06	0.44	**Dy_Tot**	0.12	0.03	0.37	0.09	0.38
**MnO_XRF**	0.004	0.001	0.01	0.002	0.01	**Er_Tot**	0.06	0.02	0.19	0.05	0.20
**MgO_XRF**	0.03	0.005	0.09	0.02	0.09	**Eu_Tot**	0.03	0.006	0.08	0.02	0.08
**CaO_XRF**	0.03	0.004	0.09	0.01	0.09	**Ga_Tot**	0.36	0.06	1.08	0.18	1.10
**Na2O_XRF**	0.01	0.01	0.04	0.04	0.06	**Gd_Tot**	0.11	0.04	0.34	0.11	0.35
**K2O_XRF**	0.04	0.01	0.11	0.04	0.11	**Ge_Tot**	0.05	0.006	0.14	0.02	0.14
**P2O5_XRF**	0.002	0.001	0.007	0.002	0.007	**Hf_Tot**	0.32	0.07	0.95	0.21	0.98
**LOI_XRF**	0.25	0.04	0.75	0.11	0.76	**Ho_Tot**	0.02	0.006	0.07	0.02	0.07
**PC1_XRF**	0.15	0.05	0.44	0.14	0.47	**La_Tot**	1.09	0.27	3.27	0.80	3.37
**PC2_XRF**	0.10	0.03	0.31	0.09	0.33	**Lu_Tot**	0.01	0.002	0.03	0.007	0.03
**PC3_XRF**	0.11	0.04	0.34	0.11	0.36	**Nb_Tot**	0.25	0.08	0.76	0.25	0.80
**PC4_XRF**	0.14	0.02	0.43	0.06	0.43	**Nd_Tot**	0.73	0.24	2.19	0.71	2.31
**PC5_XRF**	0.08	0.02	0.25	0.07	0.26	**Ni_Tot**	1.09	0.11	3.28	0.33	3.29
**PC6_XRF**	0.12	0.02	0.37	0.05	0.37	**Pb_Tot**	2.44	0.47	7.33	1.40	7.46
**PC7_XRF**	0.07	0.02	0.22	0.06	0.23	**Pr_Tot**	0.20	0.06	0.59	0.19	0.62

**PC8_XRF**	0.06	0.01	0.19	0.04	0.20
**Ba_AR**	2.97	0.60	8.90	1.81	9.08
**Be_AR**	0.03	0.004	0.09	0.01	0.09
**Ce_AR**	1.59	0.22	4.78	0.65	4.83
**Co_AR**	0.60	0.08	1.81	0.25	1.83
**Cs_AR**	0.03	0.009	0.10	0.03	0.10
**Dy_AR**	0.11	0.01	0.34	0.04	0.34
**Er_AR**	0.05	0.006	0.16	0.02	0.16
**Eu_AR**	0.04	0.004	0.12	0.01	0.12
**Fe_AR**	844	121	2530	364	2560
**La_AR**	0.65	0.10	1.95	0.30	1.97
**Mn_AR**	36.4	6.06	109	18.2	111
**Nd_AR**	0.91	0.10	2.73	0.31	2.74
**Ni_AR**	0.40	0.08	1.21	0.25	1.24
**Pb_AR**	2.43	0.59	7.28	1.77	7.49
**Rb_AR**	0.46	0.08	1.38	0.25	1.40
**Sr_AR**	0.92	0.14	2.77	0.41	2.80
**Th_AR**	0.10	0.02	0.30	0.06	0.30
**Tm_AR**	0.005	0.001	0.02	0.002	0.02
**Zn_AR**	4.42	0.45	13.3	1.34	13.3
**PC1_AR**	0.32	0.03	0.97	0.09	0.97
**PC2_AR**	0.10	0.02	0.30	0.07	0.30
**PC3_AR**	0.16	0.03	0.48	0.08	0.49
**PC4_AR**	0.17	0.03	0.50	0.08	0.51
**PC5_AR**	0.09	0.02	0.27	0.06	0.28
**PC6_AR**	0.20	0.02	0.61	0.07	0.61
**PC7_AR**	0.11	0.02	0.34	0.05	0.35
**PC8_AR**	0.10	0.01	0.30	0.04	0.30

**Rb_Tot**	3.08	0.49	9.23	1.46	9.34
**Sc_Tot**	0.27	0.05	0.82	0.15	0.83
**Sm_Tot**	0.17	0.04	0.50	0.13	0.52
**Sn_Tot**	0.10	0.02	0.30	0.06	0.31
**Sr_Tot**	1.37	0.39	4.11	1.17	4.28
**Ta_Tot**	0.02	0.009	0.07	0.03	0.07
**Tb_Tot**	0.03	0.006	0.09	0.02	0.09
**Th_Tot**	0.33	0.08	0.98	0.24	1.01
**U_Tot**	0.06	0.01	0.17	0.04	0.18
**V_Tot**	2.71	0.32	8.14	0.97	8.19
**W_Tot**	0.05	0.02	0.14	0.06	0.15
**Y_Tot**	0.69	0.16	2.06	0.47	2.11
**Yb_Tot**	0.07	0.02	0.21	0.05	0.22
**Zn_Tot**	2.01	0.47	6.03	1.42	6.19
**Zr_Tot**	15.5	2.78	46.5	8.34	47.2
**PC1_Tot**	0.75	0.10	2.24	0.31	2.26
**PC2_Tot**	0.44	0.08	1.31	0.24	1.34
**PC3_Tot**	0.61	0.05	1.82	0.16	1.82
**PC4_Tot**	0.26	0.04	0.78	0.13	0.79
**PC5_Tot**	0.41	0.05	1.23	0.16	1.24
**PC6_Tot**	0.21	0.05	0.63	0.14	0.65
**PC7_Tot**	0.12	0.05	0.35	0.14	0.38
**PC8_Tot**	0.34	0.03	1.01	0.09	1.01

REFERENCES

43. Nichols C. The mapping of Canberra urban geochemistry within a forensic context [Honours thesis]. Canberra, Australia: National Centre for Forensic Studies, University of Canberra 2018.
44. Geoscience Australia. XRF analysis using a Bruker S8Tiger. Canberra, Australia: Standard Operating Procedure, Internal Laboratory Report; 2016.
45. Geoscience Australia. Glass bead fusion. Canberra, Australia: Standard Operating Procedure, Internal Laboratory Report; 2017.
46. Geoscience Australia. Loss on ignition determination. Canberra, Australia: Standard Operating Procedure, Internal Laboratory Report; 2016.
47. Geoscience Australia. Solution preparation for ICP-MS. Canberra, Australia: Standard Operating Procedure, Internal Laboratory Report; 2018.
48. Geoscience Australia. Sample preparation for ICP-MS. Canberra, Australia: Standard Operating Procedure, Internal Laboratory Report; 2018.
49. Geoscience Australia. Instrument set up and analysis for ICP-MS. Canberra, Australia: Standard Operating Procedure, Internal Laboratory Report; 2018.
50. Hron K, Templ M, Filzmoser P. Imputation of missing values for compositional data using classical and robust methods. Comp Stat Data Anal. 2010;54:3095–107. doi: 10.1016/j.csda.2009.11.023.
51. Hornik K. The comprehensive R archive network. Wiley Interdiscipl Rev: Comp Stat. 2012;4:394–8. doi: 10.1002/wics.1212.
52. Aitchison J. The statistical analysis of compositional data. London, U.K.: Chapman & Hall; 1986. Reprinted with additional material, The Blackburn Press; 2003.
53. Campbell GP, Curran JM, Miskelly GM, Coulson S, Yaxley GM, Grunsky EC, et al. Compositional data analysis for elemental data in forensic science. Forensic Sci Int. 2009;188:81–90. doi: 10.1016/j.forsciint.2009.03.018.
54. Hanson BA, Bostock M. Package 'ChemoSpec'. Exploratory chemometrics for spectroscopy (v. 4.4.97) [computer program]. R version, 2017. https://cran.r-project.org. Accessed 2021 February 24.
55. Hengl T. Finding the right pixel size. Comp Geosci. 2006;32:1283–98. doi: 10.1016/j.cageo.2005.11.008.
56. Caritat P de, Cooper M. A continental-scale geochemical atlas for resource exploration and environmental management: The National Geochemical Survey of Australia. Geoch Expl Env Anal. 2016;16:3–13. doi: 10.1144/geochem2014-322.
57. Reimann C, Caritat P de, GEMAS Project Team, NGSA Project Team. New soil composition data for Europe and Australia: Demonstrating comparability, identifying continental-scale processes

and learning lessons for global geochemical mapping. Sci Tot Env. 2012;416:239–52. doi: 10.1016/j.scitotenv.2011.11.019.
